# COVID-19 vaccine hesitancy among adults in India: A primary study based on health behavior theories and 5C psychological antecedents model

**DOI:** 10.1371/journal.pone.0294480

**Published:** 2024-05-09

**Authors:** Sumit Aggarwal, Lucky Singh, Umaer Alam, Saurabh Sharma, Shashi Kala Saroj, Kamran Zaman, Mohd Usman, Rajni Kant, Himanshu Kumar Chaturvedi

**Affiliations:** 1 Indian Council of Medical Research- Headquarters (ICMR-Hqrs), Ansari Nagar, New Delhi, India; 2 ICMR- National Institute of Medical Statistics (ICMR-NIMS), Ansari Nagar, New Delhi, India; 3 ICMR-Regional Medical Research Centre (RMRC), Gorakhpur, Uttar Pradesh, India; 4 ICMR-National Institute of Traditional Medicine (ICMR-NITM), Belagavi Karnataka, India; University of Haifa, ISRAEL

## Abstract

Despite the significant success of India’s COVID-19 vaccination program, a sizeable proportion of the adult population remains unvaccinated or has received a single dose of the vaccine. Despite the recommendations of the Government of India for the two doses of the COVID-19 vaccine and the precautionary booster dose, many people were still hesitant towards the COVID-19 full vaccination. Hence, this study aimed to identify the primary behavioral and psychological factors contributing to vaccine hesitancy. Cross-sectional data was collected via a multi-stage sampling design by using a scheduled sample survey in the Gorakhpur district of Uttar Pradesh, India, between 15 July 2022 to 30 September 2022. This study has utilized three health behavior models—the *Health Belief Model (HBM)*, the *Theory of Planned Behavior (TPB)*, and the *5C Psychological Antecedents* of vaccination, and employed bivariate and multivariable binary logistic regression model to assess the level of vaccine hesitancy and predictive health behavior of the respondents. Results indicate that among the constructs of the HBM and 5C Antecedents models, "perceived benefits", "confidence" and "collective responsibility" showed a lesser likelihood of COVID-19 vaccine hesitancy. However, in the TPB model constructs, a ‘negative attitude towards the vaccine’ showed a four times higher likelihood of COVID-19 vaccine hesitancy. From the future policy perspective, this study suggested that addressing the issue of ‘negative attitudes towards the vaccine’ and increasing the trust or confidence for the vaccine through increasing awareness about the benefits of the vaccination in India may reduce vaccine hesitancy.

## Introduction

COVID-19 is an infectious disease caused by the severe acute respiratory syndrome coronavirus 2 (SARS-CoV-2). On the 11th of March 2020, it was declared a global pandemic by the World Health Organization (WHO) [1]. This had put a huge catastrophic impact on the health well-being, and socio-economic status of the global population, including India [2,3]. To control and manage the risk of COVID-19 infection, along with measures of social distancing, wearing masks, and nationwide lockdown, mass vaccination was also the priority of the Government of India [4,5]. Initially, under the *’National COVID-19 Vaccination Programme*’, the Government of India has introduced the ‘*COVAXIN’* and *‘COVISHIELD’* vaccines in multiple phases, from 16^th^ January 2021 onwards. The ‘*COVAXIN*’ is an inactivated virus-based vaccine (a viral vector vaccine) developed under the stewardship of the Government of India, by the Bharat Biotech (collaboratively with the Indian Council of Medical Research-National Institute of Virology, Pune), and the ‘*COVISHIELD*’ by the Serum Institute of India (Oxford-AstraZeneca) [6,7].

To maintain vaccine equity, both vaccines were provided without any cost, at the public health facilities, and with a capped price at the private health facilities to the priority population (healthcare workers, frontline workers, above 60 years of age citizens, and above 18 years old), irrespective of their socio-economic status [8,9]. Various Studies mentioned that the vaccine has made an effective reduction in the severity of the disease outcomes, including hospitalization and deaths, in the later phases of COVID-19 [10–12].

Based on the recommendations of the WHO Strategic Advisory Group of Experts on Immunization (SAGE) target of 70% vaccination by June 2022 [4,10], India has achieved three-fourths single-dose vaccine (more than 2 billion doses of vaccines, and 900 million people with complete two doses of the COVID-19 vaccine by the end of this survey (30 September 2022). However, almost one-fourth of the total population remained unvaccinated during the same period [11–13]. This gap showed that despite, the great success of the COVID-19 vaccination in India, people had concerns, and skepticism about the safety, effectiveness, and efficacy of the COVID-19 vaccine [14,15]. Despite the national and international efforts towards mass awareness campaigns and promoting the effectiveness of the COVID-19 vaccine, various studies have documented vaccine hesitancy among people who have doubts over the vaccine and the vaccination process [16,17].

World Health Organization (WHO) has identified ‘vaccine hesitancy’ as the prominent reason for the lesser vaccination coverage and listed it among the top ten threats to global public health, especially among lower and middle-income countries [18,19]. The WHO has defined vaccine hesitancy as the delay in acceptance or refusal of vaccines despite the availability of vaccination services. It is complex and context-specific which varies over time, place, and vaccines. It includes factors such as complacency, convenience, and confidence [20,21]. Since the concept of vaccine hesitancy is much explored in Western, educated, industrialized, rich, and democratic (WEIRD) countries and developed countries, compared to low- and middle-income countries (LMIC) [22], in India, there is a dearth of studies on vaccine hesitancy, especially for the COVID-19 vaccine, and its major determining factors [23].

Based on the literature survey, the ‘*HBM*’, the ‘*TPB*’, and the ‘*5C Psychological Antecedents’* of vaccination are found as the prominent theories, which can predict and explain the variations in the individuals’ health behavior outcomes (acceptance or refusal) [24,25]. The HBM assumed that adverse health conditions could be avoided among individuals by following a recommended preventive behavior. It is also assumed that future health behaviors could be predicted, explained, and identified based on existing health beliefs among people [26]. The HBM has five major constructs: (i) *perceived susceptibility* (individual’s belief about the disease susceptibility); (ii) *perceived severity* (belief about the seriousness of the disease); (iii) *perceived benefits* (belief in the usefulness of the health behavior to avert the risk of the disease), (iv) *perceived barriers (*belief in the obstacles to performing a health behavior); and (v) *cues to action* (motivational factors to practice the health behavior) [27–29]. This theory assumed that health beliefs could be collectively predicted the health behavior of an individual, but, not as a set of combinations or weights. Therefore, these five sets have been used only in their collective form to identify the level of trust of people in government initiatives and control measures to curb the pandemic [30,31].

The next model is the ‘*TPB’*, which is based on the person’s behavioral intentions to perform a given health behavior [26,32]. These intentions are assumed to capture the motivational determinants that are driven by the attitudes toward a particular behavior, the ‘subjective norm’ to the health behavior, and the perceived control over the health behavior. The combination of behavioral intentions and perceived behavioral control has resulted in variations in an individual’s behavior [33]. This model has used the three major constructs: (i) *attitudes* (individual’s assessment of the action); (ii) *subjective norms* (perceived social pressure for the behavior), and (iii) *perceived behavioral control* (combination of perceived control and self-efficacy) [27,34]. Studies showed that in the context of COVID-19 vaccination individual’s beliefs in the perceived necessity, benefit, and effectiveness of the vaccine, and their subjective norms related to their attitude towards the vaccine were determining factors [35].

In the third model of ‘*5C psychological vaccine antecedents*, studies mentioned that it is used to understand the individual’s health behavior towards the vaccine and vaccination process [36,37]. This model is an extension of the WHO SAGE Working Group’s 3C Model (2012), which includes the ‘*Confidence’*, *‘Complacency’*, and *‘Convenience’* constructs, however, in 2019, WHO formed the 5C model with the addition of the ‘*Calculation*’, and *‘Collective Responsibility’* constructs [26,35]. In this model, items of the ‘*Confidence*’ construct aim to measure the level of trust in vaccine effectiveness, safety, necessity, health delivery system, and competency of health professionals and healthcare services. The ‘*Complacency*’ construct focuses on the assessment of the perception of the level of the risk of disease and the necessity of the vaccination. Whereas, the *‘Constraints’* items estimate the level of barriers to the availability, accessibility, and affordability of vaccination. Additionally, the ‘*Calculation’* items assessed the individual’s motivation and perception of the advantages and disadvantages of vaccination, and the ‘*Collective responsibility’* construct aims to examine the perception of social or community responsibility with a socially empathetic behavior or to attain ‘*herd immunity’* [38,39].

These models are not much explored in the Indian health behavior context, especially for the COVID-19 vaccine [40–42]. Additionally, India is one of the largest populated countries at the global level [43], and among its states, Uttar Pradesh (U.P.) shared the largest contribution to the overall population [44,45]. Furthermore, the U.P. has achieved remarkable success in the government-driven COVID-19 vaccination awareness and campaigns, among all 75 districts. Gorakhpur district, which was among the top five districts in the initial phases of vaccination, coverage, is emerging as a significant district at the state as well as national levels [46]. To represent the rural as well as urban population with socio-economic, and demographic dynamic characteristics, Gorakhpur district was taken as the study area [22,44,47].

From, a future perspective, this study is crucial to understanding the level of vaccine hesitancy and behavioral determinants by taking it as an example for better preparedness for any short-term variations in the COVID-19 infections, or any other disease outbreak in the near future. Therefore, this study has aimed to analyze the level of COVID-19 vaccine hesitancy by the background characteristics of the population and to understand the behavioral and psychological factors of vaccine hesitancy, by using the HBM, TPB, and 5C antecedents models.

## Materials and methods

### Study setting, design, and sampling

The vaccine hesitancy study is a population-based cross-sectional study that collected data from the two blocks (i.e. ‘*Charganwa*’ and ‘*Bhathat*’) of the Gorakhpur district of Uttar Pradesh (U.P.), India, between July 2022 and September 2022. According to the Census of India [2011], the total population of *Gorakhpur* district was 4,440,895, and among them, men and women were 51.29% and 48.71%, respectively [48]. Almost four-fifths of the total population resided in rural households (81.17%), and 18.82% in urban households. Further, at the administrative level, Gorakhpur is subdivided into 7 sub-divisions (tehsils), 19 development blocks, and 84 villages. Among them, the Gorakhpur sub-division has 32 villages (the highest number of villages), and almost two-fifths (44.35%) of the Gorakhpur sub-division population is urban, as compared to other tehsils. Further, among the five blocks of the Gorakhpur sub-division, the ‘*Charganwa*’ block represented the ‘highest’ coverage area for the COVID-19 vaccine, and the ‘*Bhathat*’ block represented the ‘lowest’ coverage area for the COVID-19 vaccination, in the Gorakhpur district. This low coverage of vaccine was more prominent among the rural households, while high coverage was found among the urban households,. The data was taken from the block administrative officer, which were contacted by the administration of the institute, according to the advisory of the Ministry of Health and Family Welfare [49].

In the four stages of the multi-stage cross-sectional sampling method, the 600 adult respondents (≥18 years of age) were interviewed between July 15, 2022, and September 30, 2022. **I**n stage 1, the entire Gorakhpur district was subdivided into seven tehsils, and one tehsil (Gorakhpur Sadar) was selected, which has almost equal representation of the rural and urban population. In Stage 2: Among the five blocks of the Gorakhpur Sadar, two blocks were selected as the lowest and highest COVID-19 vaccine coverage area, at the time of the survey. In stage 3: the villages were stratified by the village population size, and the stratum of large and small villages was done as per the census data. Lastly, in Stage 4, villages were selected by a simple random sampling method from each stratum. In each village, a total of adult men and women who were not vaccinated or had a single dose of vaccination were identified with the help of the local health care worker or ANM. A detailed description of the study was shared in the local (Hindi) language, and consent was obtained before the interview. Those, who agreed and provided their consent were included in this study.

Those who were fully vaccinated (had two or more doses of the COVID-19 vaccine) were not included in this survey. The sample size was calculated, by using the following formula:

n=(Z_α^2*p*q*(1+R)*(deff))/d^2

Where *n* denotes the estimated sample size (600 samples); *α* = the level of statistical significance that was set at 0.05; Zα = the z value at 95% confidence level, (Here, *zα* = 1.96, with 95% confidence level); d denotes the margin of error [50]. Here, d = .05; p is the prevalence of vaccine hesitancy to be 34.3%; q = 1-p (q = 65.7%); R response rate (Here, R = .1); deff denotes the design effect (here, deff is 1.5) [51].

The data was collected by using ‘*The Kobo Toolbox Platform’* which is available in the public domain. The quality of the data, real-time, and location were continuously checked through its online connectivity at the website **http://www.kobotoolbox.org/** [52]. The ethical clearance were given by the authors’ institute, in New Delhi, India. Ethical approvals were sought from both authors’ institutes.

### Survey measures

The samples were collected through personal interviews using the structured questionnaire, which was developed by the study investigators. Before the conduct of the interview, the participants were well-informed about the survey, and informed consent was sought by the interviewers. The questionnaire was available in Hindi and English languages. Three interviewers had collected the data and were well-qualified and trained for the primary survey. The structured questionnaire has focused on the major components of the HBM, TPB, and 5C psychological antecedents models, along with the socioeconomic and demographic details of the respondents. The questionnaire was divided into two major parts ‘A’ and ‘B’. Part A collected the information of the respondents, while Part B was further divided into eight sections, which collected the study-related information covered under multiple eight sections. Section 1 dealt with the participant’s socioeconomic and demographic characteristics, Section 2 collected information on the COVID-19-related history (disease/ infections/ deaths); Section 3 related to the ‘Knowledge or perception about the COVID-19 vaccine and vaccine hesitancy’; and Section 4 and 5 asked about the respondent’s ‘health status’. In addition, to understand the respondent’s attitude, subjective norms, perceived belief and behavior, and anticipated regret, this study has included the components of the ‘Health Belief Model’, ‘Theory of Planned Behavior’, and ‘The 5C psychological antecedents of vaccination’, in section 5, 6, and 7, respectively. However, the 8^th^ Section deals with the ‘*Knowledge and Belief regarding the COVID-19 vaccination*.’

In the HBM section, items were included based on the five components of the model, i.e., ‘*perceived susceptibility*’ ‘*perceived severity* (∝ = **0.781**),’, ‘*perceived benefits* (∝ = **0.788**)’, ‘*perceived barriers* (∝ = **0.626**)’, and ‘c*ues to action’*. Except for ‘Cues to Action’, the rest of the components were rated on a five-point ‘*Likert Scale’* ranging from ‘Strongly Disagree’ (1) ‘Disagree’ (2), ‘Can’t Say Anything’ (3), ‘Agree’ (4), ‘Strongly Agree’ (5). Whereas, ‘Cues to Action’ was dichotomized into ‘*Yes*’ or ‘*No*’. Furthermore, in the TPB model, the responses ratings were given on the five-point Likert scale (‘Strongly Disagree’, ‘Disagree’, ‘Can’t say Anything’, ‘Agree’, and ‘Strongly Agree’) for each item of the four components of TPB: ‘*negative attitude towards vaccine*’ (∝ = **0.781**), ‘*subjective norm*’, ‘*perceived behavioral control*’, and ‘*anticipated regret’*. Similarly, in the 5C psychological antecedents of the COVID-19 vaccination model, the same ratings were used to measure the 14 items of the five components of 5C: (a) Confidence (∝ = **0.844**), (b) Constraints, (c) Complacency, (∝ = **0.637**), (d) calculation, (∝ = **0.864**, and (5) Collective Responsibility (∝ = **0.637**). A detailed summary of the items in these three models has been given in the S1 Table in S3 File, along with their reliability coefficient for each component.

To measure vaccine hesitancy, ‘Question (3.8): Are you planning to take the COVID-19 vaccine that is currently available?’ was asked to the respondents, with five-point Likert scale response, i.e., ‘*Definitely*’, ‘*Probably*’, ‘*Not sure’*, ‘*Probably not’*, and ‘*Definitely not’*. Among the five-point Likert scales, ‘*Definitely*’ and ‘*Probably*’ responses were considered non-hesitant responses, on the other hand, ‘*Not Sure*’, ‘*Probably not*’, and ‘*Definitely not*’ responses were considered vaccine-hesitant.

### Statistical analyses

Descriptive statistics were used to analyze the socioeconomic, and demographic characteristics and knowledge of COVID-19 and its vaccine. The sample distribution and percentage for the background characteristics were measured by the mean with standard deviation (SD). However, due to the skewed nature of the samples among the ‘*age*’ and ‘households’ members’, the median with SD was calculated to know the distribution. However, bivariate analyses were used to estimate the level of vaccine hesitancy based on background characteristics, knowledge level about COVID-19 vaccination, and intention of getting COVID-19 vaccination. Moreover, the *Chi-square test* was used to compare the observed results with the expected results, with p-values (two-tailed with a significance level of 5%). Similarly, the bivariate analyses were performed for the three models (HBM, TPB, and 5C), separately, to assess the level of COVID-19 vaccine hesitancy by the items of the models. The details of the outcome variable, i.e., vaccine hesitancy are mentioned above. The *multivariable binary logistic regression* model was employed to predict the association between the level of COVID-19 vaccine hesitancy and the major components of the HBM, TPB, and 5C psychological antecedent models. All three health behavior models were analyzed separately, by using the *STATA-15* software [53].

## Results

### Demographic and socio-economic characteristics of the sample

Table 1 indicates the sample distribution and the percentage of COVID-19 vaccine hesitancy based on the socio-economic characteristics of the selected respondents (N = 600) in this study. In this study, 524 respondents (87.3%) had one dose of COVID-19, while, 76 respondents (12.7%) were unvaccinated at the time of this survey (July 2022 to September 2022). Almost half (n = 304) of the respondents were female (50.7%). Most of the respondents (185) belonged to the 18–24 years age group (30.8%), followed by the 25–34 years age group (n = 139, 23.2%), and the 35–44 years age group (n = 107, 17.8%), while, 55 and above years age group had the least representation in this survey (n = 78, 13%). The median age of the samples was 32 years ± 14.73. Almost, 431 (71.8%) respondents were married, and 169 respondents (28.2%) were unmarried during the survey. Similarly, by educational status, most of the respondents were uneducated or had no education (n = 150, 25%) or primary or secondary (n = 179, 29.8%) level educated; and only 41 (6.8%) respondents were undergraduate or above. The median number of household members was 6 ±4.04. Other details are given in Table 1.

**Table 1:** Sample distribution and percentage of COVID-19 vaccine hesitancy by the respondent’s background characteristics (n = 600).

Variables	Study sample (n)	Percentage (%)	Hesitancy (%)
**Age**			χ2 = 11.8285, p = 0.037
18–24	185	30.8	9.2
25–34	139	23.2	8.6
35–44	107	17.8	13.1
45–54	91	15.2	13.2
55–64	45	7.5	17.8
65 and above	33	5.5	27.3
**Age, median (SD)**		32 (14.73)	
**Sex**			χ2 = 0.765, p = 0.38
Male	296	49.3	13.2
Female	304	50.7	10.9
**Marital status**			χ2 = 0.406, p = 0.52
Never married	169	28.2	10.7
Ever married	431	71.8	12.5
**Education status**			χ2 = 11.762, p = 0.04
No education	150	25	18
Below Primary	33	5.5	21.2
Primary and secondary education	179	29.8	7.8
Senior Secondary	104	17.3	9.6
Intermediate	93	15.5	9.7
Undergraduate and above	41	6.8	12.2
**Occupation**			χ2 = 20.945, p = 0
Unemployed	260	43.3	8.8
Student	84	14	11.9
Employed (Formal sector)	81	13.5	27.2
Employed (Informal sector)	175	29.2	9.7
**Financial Status**			χ2 = 0.031, p = 0.86
Medium/High	239	39.8	11.7
Low	361	60.2	12.2
**Caste**			χ2 = 6.123, p = 0.05
General	33	5.5	21.2
OBC	356	59.3	9.6
SC/ST	211	35.2	14.7
**Household members, median (SD)**		6 (4.04)	
**Any 60 or above years old member in the family**			χ2 = 4.1156; p = 0.042
No	420	70	10.2
Yes	180	30	16.1
**Tobacco use**			χ2 = 0.833, p = 0.36
Never/ Former	459	76.5	11.3
Current	141	23.5	14.2
**Alcohol use**			χ2 = 2.701, p = 0.1
Never/ Former	455	75.8	10.8
Current	145	24.2	15.9
**Household health insurance**			χ2 = 0.124, p = 0.73
No	468	78	11.8
Yes	132	22	12.9
**Covid doses (July 2022 to September 2022)**			χ2 = 35.979, p = 0.00
None	76	12.7	32.9
One dose	524	87.3	9
**Health care worker**			χ2 = 0.125, p = 0.72
No	594	99	12
Yes	6	1	16.7
**Total**	**600**	**100**	**12**

Other details regarding the respondent’s knowledge about the COVID-19 vaccine; vaccination process; behavioral practices to prevent COVID-19; and conspiracy belief regarding the COVID-19 vaccine; and items used in the HBM, TPB, and 5C psychological antecedents of vaccination models, are given in the S2 to S9 Tables in S3 File.

### COVID-19 vaccine hesitancy by respondent’s background characteristics

The study showed that 32.9% of unvaccinated people were hesitant about the COVID-19 vaccine compared to those who had a single dose of the COVID-19 vaccine (9%) (Table 1). Moreover, by socio-economic and demographic characteristics, older (65+) people (27.3%), men (13.2%), married persons (12.5%), below primary level educated (21.2%), and those with no education (18%), employed in the formal sector (27.2%) had a comparatively higher hesitancy for the COVID-19 vaccine than their counterparts. Similarly, amongst the social groups, respondents belonging to the general caste (21.2%), and having older family members (16.1%,), had higher hesitancy than the other caste groups. Similarly, respondents who were regularly consuming alcohol (15.9%) and tobacco (14.2%) (in any form) showed higher vaccine hesitancy than their counterparts.

### Vaccine hesitancy by source of information about the COVID-19 vaccine and vaccination process

**Fig 1:**
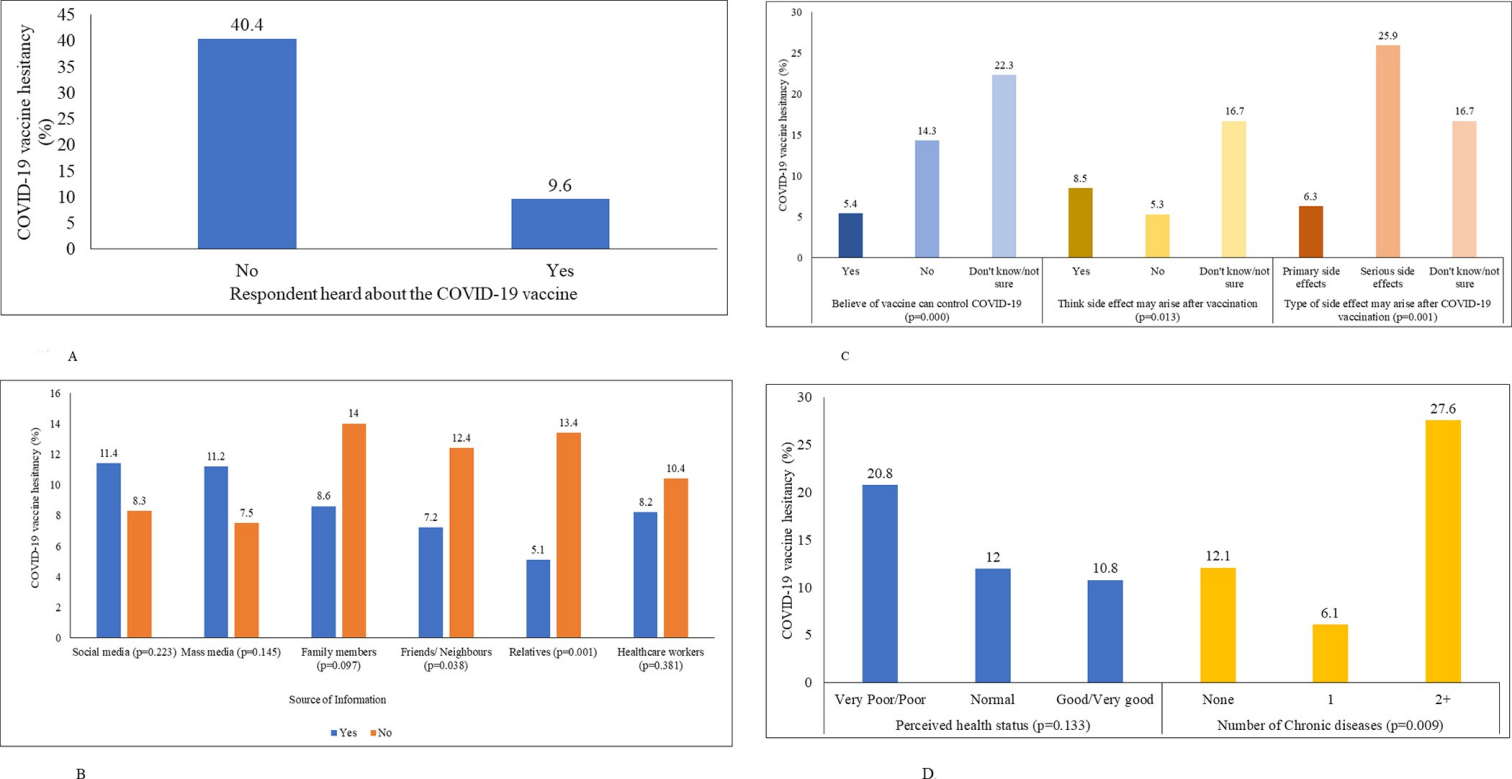
A. COVID-19 vaccine hesitancy by the respondent’s knowledge about the COVID-19 vaccine, (N = 600). B. COVID-19 vaccine hesitancy by the respondent’s source of information about the COVID-19 vaccine. C. COVID-19 vaccine hesitancy by the respondent’s belief and thinking about the COVID-19 vaccine and its side-effects. D. COVID-19 vaccine hesitancy by the respondent’s health status.

Almost 92% of respondents mentioned that they had heard about the COVID-19 vaccine through multiple sources of information. Among them, the major sources of information were family members (81.90%), mass media (56.60%), friends and neighbors (55%), nearest relatives (46.10%), social media (41.20%), and health workers (37.60%), consecutively. In Fig 1B, by the source of information on the COVID-19 vaccine, those who had information from, a friend or neighbor (7.2%), or relatives (5.1%), showed significantly less vaccine hesitancy than those who did not have it (S3 Table in S3 File).

In addition, 47.2% mentioned that only one or two doses are enough of the COVID-19 vaccine, whereas, 6.7% of individuals didn’t know the required number of the doses of the COVID-19 vaccine. Interestingly, 381 (68.9%) individuals didn’t know the duration of the effectiveness of the COVID-19 vaccine (S3 Table in S3 File). Almost 404 (73.1%) individuals believe that the COVID-19 vaccine could prevent COVID-19. More than two-thirds of the respondents (n = 351,63.5%) had fear about the side effects of the vaccine. Among them, 300 respondents (85.5%) feared primary side effects, 27 individuals (7.7%) had a fear of secondary side effects, and 24 respondents (6.8%), did not know the side effects. Nearly 111 (19%) respondents were suffering from one (13.7%) or more (4.8%) chronic diseases. Vaccine hesitancy was higher among individuals who were not sure about the effectiveness of the vaccine and were either suffering from some chronic ailments (Fig 1C and 1D).

**Fig 2:**
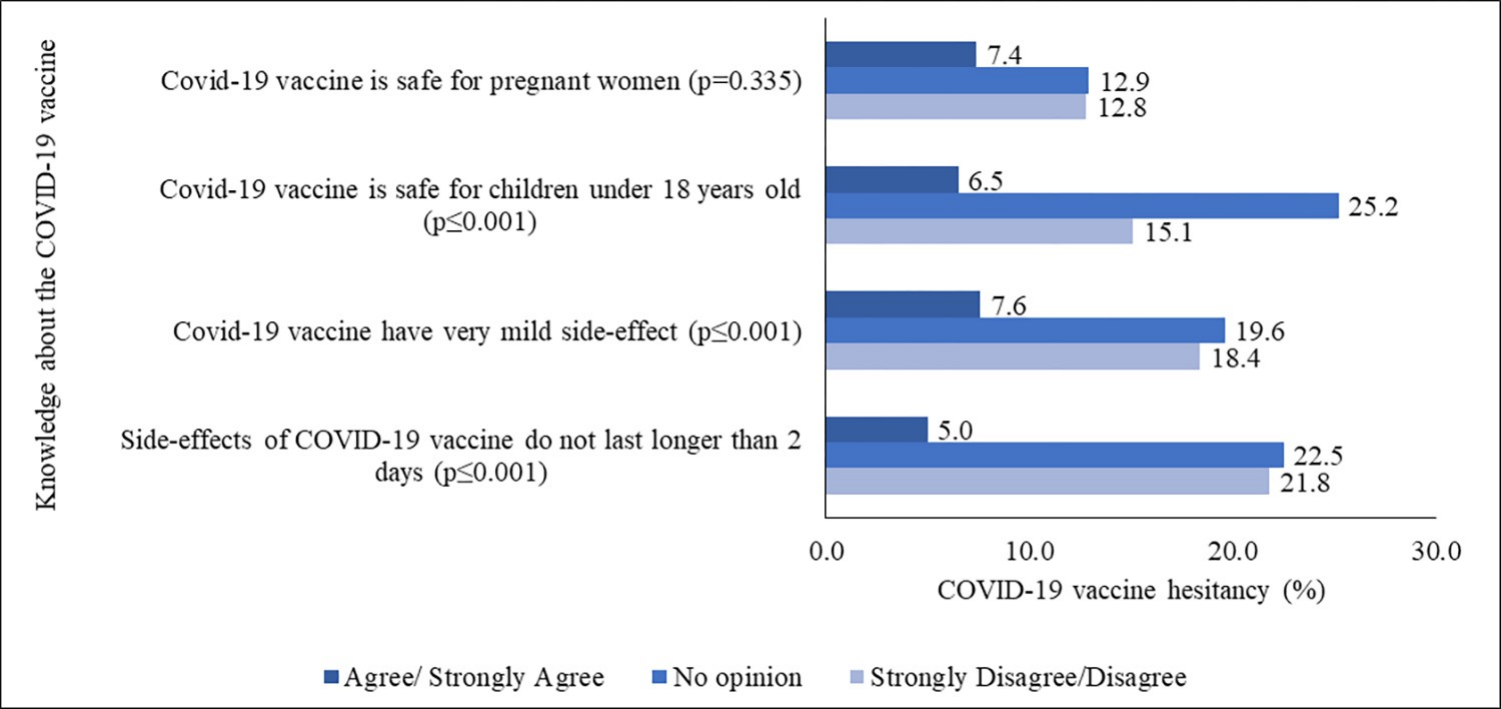
COVID-19 vaccine hesitancy (%) by knowledge about the COVID-19 vaccine.

Fig 2 and S4 Table in S3 File show that those respondents, who strongly disagreed or disagreed, or had no opinion on the asked questions regarding the knowledge about the COVID-19 vaccine (side-effect duration, level of side-effect, or safety of the COVID-19 vaccine for under-18 children and pregnant women), showed higher hesitancy for the COVID-19 vaccine, compare to those who strongly agreed or only agreed with the asked questions. In addition, 64.3% of respondents stated that they did not know that they would have to consult with a doctor, and 42.8% of respondents did not know the online registration process, to receive a COVID-19 vaccine.

**Fig 3:**
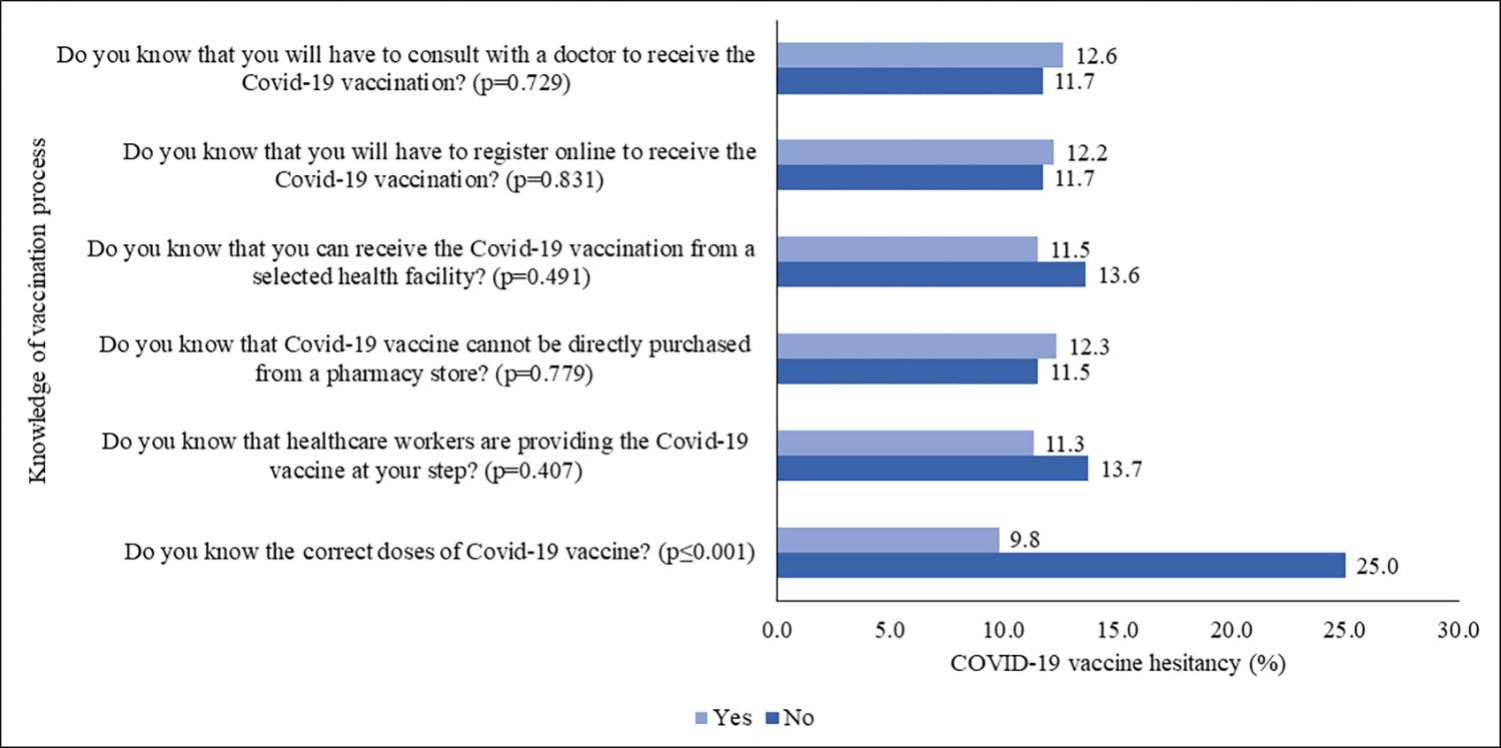
COVID-19 vaccine hesitancy (%) by knowledge about the COVID-19 vaccination process.

However, 14.7% of the respondents, who didn’t know the correct doses of the COVID-19 vaccine, had 25.0% more hesitancy than those who were aware (9.8%). Furthermore, 30.5% of respondents didn’t know that healthcare workers were providing the COVID-19 vaccine at their doorstep; 32.0% did not know that they could not purchase the vaccine from the drug-store; 24.5% did not know that they could receive the vaccine from a selected health facility, and 42.8% of individuals didn’t know the online registration method to get the COVID-19 vaccine. However, 35.7% of individuals found it important to consult a doctor before getting the COVID-19 vaccine (S5 Table in S3 File). These individuals displayed higher vaccine hesitancy than their counterparts (Fig 3).

### Vaccine hesitancy through behavioral practices to prevent COVID-19

**Fig 4:**
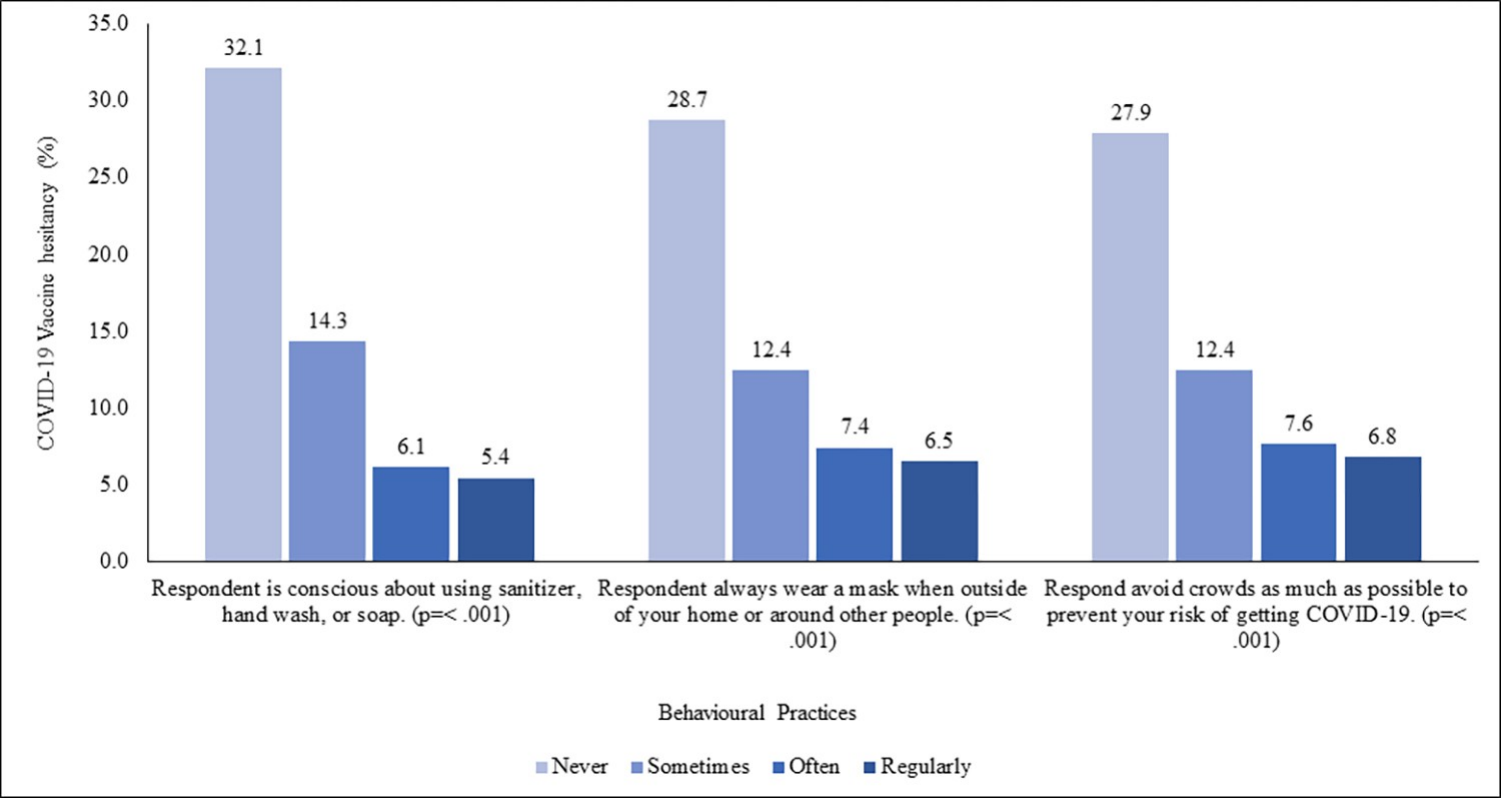
COVID-19 vaccine hesitancy (%) by the behavioral practices to prevent COVID-19.

Behavioral practices items (α = 0.886), showed that 21.7% regularly practiced handwashing behavior, and 13.8% of individuals mentioned that they don’t wear a mask in public or around people (S6 Table in S3 File). Fig 4 showed that those, who never, practiced any preventive behaviors had significantly higher vaccine hesitancy (32.1%, 28.7%, and 27.9%, consecutively), while least among those who practiced regularly (5.4%, 6.5%, and 6.8%, respectively).

### Vaccine hesitancy due to conspiracy beliefs regarding the COVID-19 vaccine

**Fig 5:**
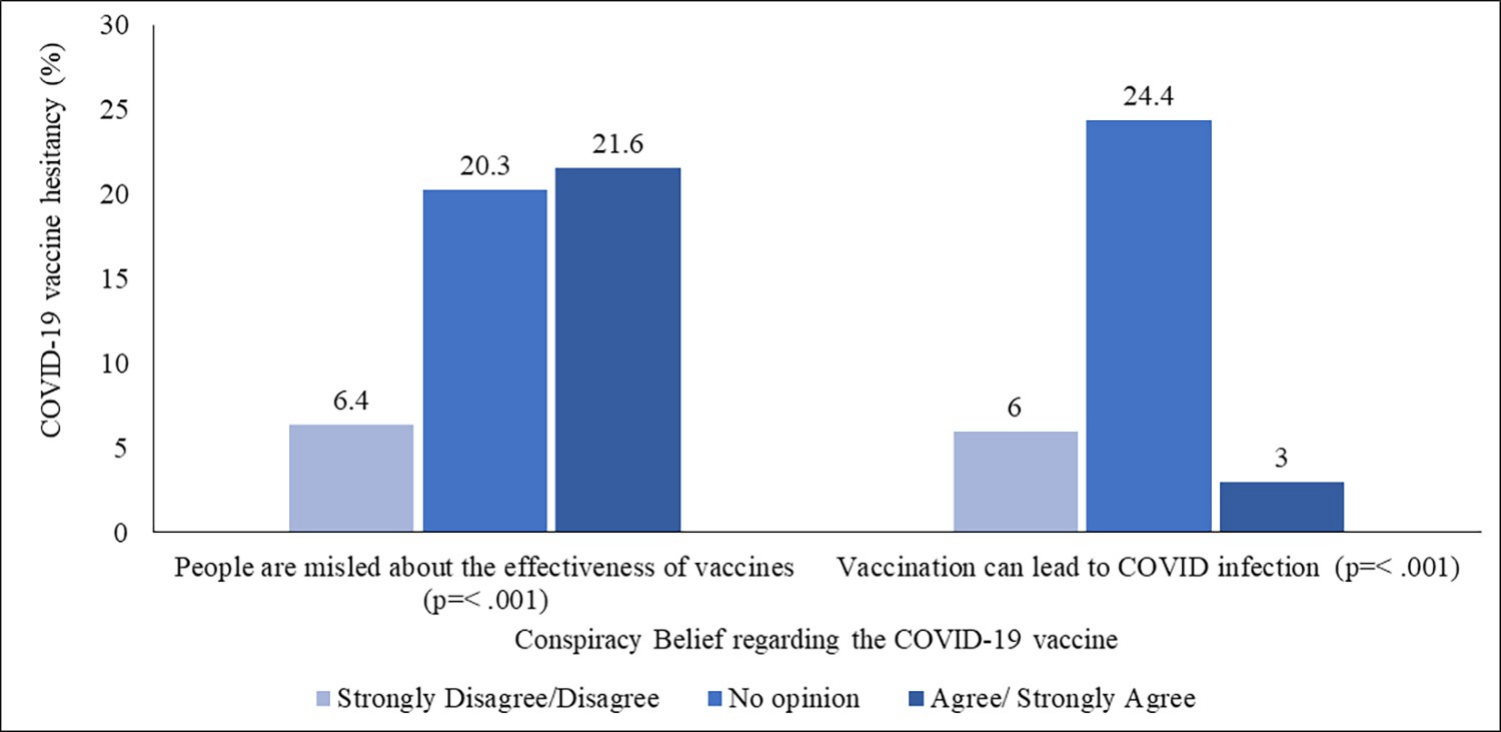
COVID-19 vaccine hesitancy (%) by the conspiracy belief regarding the COVID-19 vaccine.

Almost three-fifths of the total respondents (60.3%) disagreed or strongly disagreed with the statement that people are misled about the effectiveness of the COVID-19 vaccine, whereas, sixty-one percent believed that COVID-19 vaccination could lead to COVID-19 infection, (S7 Table in S3 File). Fig 5 displayed that COVID-19 vaccine hesitancy was higher among the people who ‘agreed or strongly agreed’ (21.6%, and 3.0%), or gave ‘no opinion’ (20.3%, and 24.4%) on the statements.

**Fig 6:**
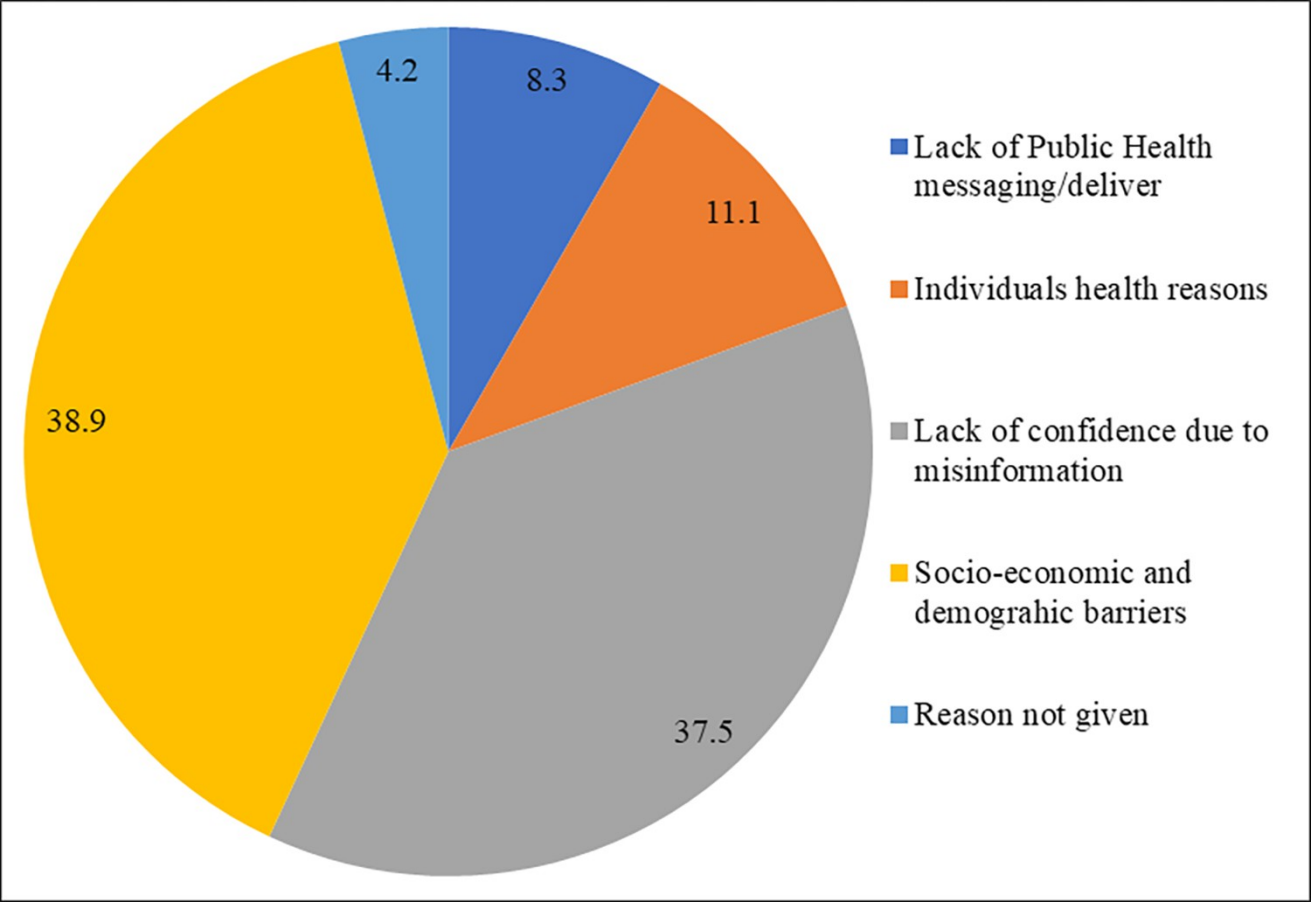
Major reasons for the COVID-19 vaccine hesitancy among people who were not planning for any dose of the COVID-19 vaccine.

Fig 6 displayed the major reasons given by the respondents for vaccine hesitancy or delay in the vaccination against COVID-19. More than two-thirds of respondents’ major reasons for not getting the COVID-19 vaccine were ‘*socio-economic and demographic barriers*’ (38.9%), and ‘*lack of confidence due to misinformation*’ (37.5%).

In its continuity, by using three public health behavior theoretical models, this study provides more clarity on the major predictive behavior and barriers to COVID-19 vaccination.

### COVID-19 vaccine hesitancy by the HBM

Overall results showed that most of the individuals ‘strongly disagreed or disagreed’ with the items related to ‘perceived susceptibility’, ‘perceived severity’, and ‘perceived barriers’, except ‘perceived benefits’, or were given ‘no opinion’. However, more than three-fifths of the respondents agreed or strongly agreed with the statements related to the ‘perceived benefits’ (S8 Table in S3 File). In the component ‘Cues to Actions’, 42% of individuals reported ‘Social Media’ (Facebook, Twitter, Instagram) or online news portals as their source of knowledge for the COVID-19 vaccine, while, 59% mentioned the printed newspaper as their source of knowledge for the COVID-19 vaccine.

COVID-19 vaccine hesitancy was higher among those individuals who had given ‘No Opinion’ against the items across the components, followed by ‘strongly disagreed, or disagreed’ respondents (Table 2). Whereas, those who ‘agreed or strongly agreed’ with the items of perceived benefits showed the least vaccine hesitancy across the components of the HBM. However, the percentage of vaccine hesitancy was higher among the responses with ‘no opinion’, followed by ‘agreed or strongly agreed’ with the statements of perceived barriers, especially for the statement “Respondent concerned about the long-term side effects of the COVID-19 vaccination”, where, 24.1% with ‘no opinion’, and 11.2% with ‘agreed or strongly agreed’ responses, revealed higher hesitation than 6% with the ‘strongly disagreed/ agreed’, to get the COVID-19 vaccine. Furthermore, those respondents, who did not have the printed newspaper as their source of knowledge for the COVID-19 vaccine, revealed higher vaccine hesitancy (13.8%), than those, who had printed newspapers (10.7%). On the contrary, vaccine hesitancy is similar among social media or online news portal users and those, who didn’t have it (12.0%).

**Table 2:** Vaccine hesitancy (%) by HBM among 600 samples.

Variables	Strongly Disagree/Disagree	No opinion	Agree/ Strongly Agree	χ2	p-value
**Perceived Susceptibility**					
Respondent at high risk of COVID-19 because of his/ her health conditions	11.70	23.00	6.00	22.021	0.00
**Perceived Severity**					
Respondent will be very sick if he/she get infected by COVID-19	15.50	12.80	6.90	7.535	0.02
Respondent was very concerned that he/she could die from COVID-19	12.40	14.80	5.20	5.633	0.06
**Perceived Benefits**					
Respondent think vaccination is good because it will make him/her less worried about COVID-19	23.90	32.80	3.70	86.155	0.00
Respondent believe vaccination will decrease his/her risk of getting infected by COVID-19	23.90	30.20	5.20	65.530	0.00
Respondent think the complications of COVID-19 will decrease if he/she get vaccinated and then get infected with the Coronavirus.	15.30	30.10	4.80	65.408	0.00
**Perceived Barriers**					
Respondent worried that the possible side effects of the COVID-19 vaccination would interfere with his/her usual activities	8.20	16.40	10.50	6.592	0.04
Respondent concerned about the efficacy of the COVID-19 vaccine	9.10	22.20	9.00	17.238	0.00
Respondent have a concern that he/she may receive faulty/fake COVID-19 vaccine	6.90	19.90	10.20	20.030	0.00
It concerns respondent that the development of a COVID-19 vaccine is too rushed to test its safety properly	6.40	21.60	11.10	25.232	0.00
Respondent concerned about the long-term side effects of the COVID-19 vaccination	6.00	24.10	11.20	29.562	0.00
**Cues to Action**	**Percentage**				
Social media (e.g., Facebook) or online news portals/blogs as a source of knowledge about the COVID-19 vaccine				0.0009	0.976
Yes	12.0				
No	12.0				
Printed newspaper as a source of knowledge about the COVID-19 vaccine				1.3095	0.252
Yes	10.7				
No	13.8				

#### COVID-19 vaccine hesitancy by the TPB model

The percentages of responses given for the statements of the four constructs of the TPB reveal that the percentage of ’Strongly Disagree/Disagree’ is higher for the statements about the attitude towards the vaccine. Meanwhile, the percentage of ’agreed or strongly agreed’ is higher for the statements about the subjective norm, perceived behavioral control, and anticipated regret (S9 Table in S3 File). Moreover, the overall results presented in Table 3 show that the percentage of vaccine hesitancy is higher among those who had ’no opinion’, followed by those who ’agreed or strongly agreed’ with the statements of the constructs of the TPB model, except for the responses to the statement of anticipated regret.

**Table 3:** Vaccine hesitancy (%) by items of the TPB Model among 600 samples.

Variables	Strongly Disagree/Disagree	No opinion	Agree/Strongly Agree	χ2	p-value
**Negative Attitude toward vaccine**					
Respondent thinks the COVID-19 vaccine probably will not work	5.3	26.6	10.3	50.354	< .001
Respondent doesn’t trust the COVID-19 vaccine	5.8	37.9	13.7	81.472	< .001
Respondent thinks the COVID-19 vaccine is unnecessary	6.1	41.2	20	86.575	< .001
Respondent thinks that it is not important to get a vaccine to protect people from the COVID-19	5.8	38.5	12	85.041	< .001
Respondent does not need a COVID-19 vaccine because he/she is healthy and at low risk for infection	5.6	31.1	11.7	60.461	< .001
Respondent does not need a COVID-19 vaccine because even if he/she get infected, they will not become seriously ill	6.7	25.2	11.1	35.392	< .001
**Subjective norm**					
Respondent believes that his/her family members will support him/her to get vaccinated against COVID-19	7.3	31.3	7.4	50.207	< .001
**Perceived behavioral control**					
If respondent want, he/she can register for COVID 19 vaccination	8	25.8	8.3	29.269	< .001
**Anticipated regret**					
If respondent does not get a COVID-19 vaccine and end up getting Coronavirus, he/she will regret not getting the vaccination	17.3	37.6	6.7	66.670	< .001

Almost 37.6% of the respondents had ’no opinion’, followed by 17.3% who ’strongly disagreed or disagreed’ with the statement ’if the respondent does not get a COVID-19 vaccine and ends up getting Coronavirus, he or she will regret not getting the vaccination’ of the anticipated regret construct.

#### COVID-19 vaccine hesitancy by the *5C* psychological antecedents

According to the 5C psychological antecedents model, the statements related to confidence, calculation, and collective responsibility received higher ’agree or strongly agree’ responses, while the statements related to constraints and complacency received higher ’strongly disagreed or disagreed’ responses (S10 Table in S3 File). However, the bivariate analysis (Table 4) indicated that respondents who had ’no opinion’ for items in all five constructs exhibited higher vaccine hesitancy, followed by those who responded with ’strongly disagree or disagree’ to the statements in confidence, constraints, calculation, and collective responsibility. Notably, in the complacency construct, respondents who ’agreed or strongly agreed’ had higher hesitancy rates for the COVID-19 vaccine.

**Table 4:** Percentage of COVID-19 vaccine hesitancy by the items of 5C psychological antecedents of vaccination model among 600 samples.

Variables	Strongly Disagree/Disagree	No opinion	Agree/ Strongly Agree	χ2	p-value
**The 5C Psychological Antecedents of Vaccination**					
**Confidence**					
Respondent is completely confident that COVID 19 vaccines are safe	24.5	22.6	6.1	37.113	< .001
Respondent is completely confident that COVID 19 vaccines are effective	32.4	24.3	6.3	47.031	< .001
**Constraints**					< .001
Everyday work stress may prevent the respondent from getting vaccinated	10.8	26.4	4.1	40.942	< .001
**Complacency**					< .001
Respondent thinks that it is unnecessary to receive vaccinations as it cannot prevent COVID-19	6.7	29.5	15.5	44.200	< .001
Respondent believe that his/her immune system is powerful; it will protect him/her from COVID-19	8.3	19.1	8.4	14.829	0.001
Respondent believe COVID-19 is not much a severe disease that he/she should get vaccinated against it	6.8	29.8	9.5	45.884	< .001
**Calculation**					
When respondent thinks about getting vaccinated against COVID 19, he/she weigh the benefits and risks to make the best decision possible	10.1	33.3	6.3	59.841	< .001
When respondent thinks about getting vaccinated against COVID 19, he/she will first consider whether it is effective or not	9	31	7.2	47.695	< .001
Before get COVID-19 vaccinated, respondent need to know about this vaccine in details	10.1	30.5	8.3	36.887	< .001
**Collective responsibility**					
Respondent will take COVID 19 vaccine because, in that way, he/she can protect people with a weaker immune system	22.2	23.1	5.4	41.171	< .001
Respondent think vaccination against COVID 19 is a collective action to prevent the spread of diseases	15.2	30.6	6.5	52.556	< .001

#### Prediction of COVID-19 vaccine hesitancy by *HBM*, *TPB*, *and 5C psychological antecedents* models

Vaccine hesitancy was reported among 12% of the total respondents (N = 600). In addition, Table 5 indicates the results for the level of predictability of the association between COVID-19 vaccine hesitancy through the health behavior models (HBM, TPB, and 5C Psychological Antecedents, separately). It revealed that among all the five constructs of the HBM (perceived susceptibility, perceived severity, perceived benefits, perceived barriers, and cues to action), only the ‘perceived benefits’ showed a significant negative association with COVID-19 vaccine hesitancy [Adjusted odds ratio: 0.26, with 95% C.I.: 0.15–0.47].

**Table 5:** Multivariable binary logistics regression model for the vaccine hesitancy prediction by HBM, TPB, and 5C Psychological antecedents.

Variables	Adjusted odds ratio	p-value	95%CI	
**Model A: LR chi2(25) = 130.21**				
**HBM**				
Perceived Susceptibility	0.80	0.13	0.61	1.07
Perceived Severity	0.88	0.62	0.52	1.47
Perceived Benefits	0.26	0.00	0.15	0.47
Perceived Barriers	1.56	0.21	0.77	3.14
**Cues to Action**				
Social media (e.g., Facebook) or online news portals/blogs as a source of knowledge about the COVID-19 vaccine				
Yes (ref)				
No	0.72	0.43	0.31	1.64
Printed newspaper as a source of knowledge about the COVID-19 vaccine				
Yes (ref)				
No	1.97	0.09	0.91	4.27
**Model B: LR chi2(25) = 118.07**				
**TPB**				
Negative Attitude toward vaccine	4.17	0.00	2.06	8.45
Subjective norm	0.95	0.85	0.58	1.57
Perceived behavioral control	1.05	0.84	0.64	1.72
Anticipated regret	0.76	0.24	0.47	1.21
**Model C: LR chi2(26) = 127.80**				
**The 5c Psychological Antecedents of Vaccination**				
Confidence	0.42	0.00	0.24	0.72
Constraints	0.69	0.09	0.45	1.06
Complacency	1.60	0.11	0.90	2.84
Calculation	1.00	0.99	0.57	1.73
Collective responsibility	0.48	0.02	0.27	0.89

Note: In models A, B, and C, the effects of the following variables were controlled for all models, separately: age of the respondents, educational status, occupation, caste, any 60+ family member, number of chronic diseases, knowledge about the COVID-19 vaccine, knowledge about the COVID-19 vaccination process, behavioral practices to prevent COVID-19, conspiracy belief regarding COVID-19 vaccine.

Similarly, in the TPB model, those who had a higher negative attitude towards the COVID-19 vaccine were four times more likely to have hesitancy than those who didn’t have a less negative attitude towards the vaccine [Adjusted odds ratio: 4.17, with 95% C.I.: 2.06–8.45].

Furthermore, in the 5C model, ‘confidence’ [Adjusted odds ratio: 0.42, with 95% C.I.: 0.24–0.72], and ‘collective responsibility’ [Adjusted odds ratio: 0.48, with 95% C.I.: 0.27–0.89] components showed a significant negative association with COVID-19 vaccine hesitancy. In other words, it could suggest that a significant increase in the confidence of people in the COVID-19 vaccination, and their feeling of responsibility for society to prevent the spread of disease reduced COVID-19 vaccine hesitancy.

## Discussion

The objective of this study was to assess the level of COVID-19 vaccine hesitancy and identify the major determinants of health behaviors among the adult population of Gorakhpur district, Uttar Pradesh, India [44]. The results showed approximately 12% vaccine hesitancy among the selected participants, who were unvaccinated or had only received a single dose of the COVID-19 vaccine, at the time of this survey. This study identified the major predictive health behavior for COVID-19 vaccine hesitancy by using the three health behavior models. This study has also identified the major barriers mentioned by the respondents to getting the COVID-19 vaccines.

The findings of the study exhibited that vaccine hesitancy was higher among older individuals (> 40 years of age), mostly among married individuals, than their respective counterparts. Similarly, individuals working in the formal sector showed more vaccine hesitancy than students and employees in the informal sector [54,55]. On the contrary, respondents with below primary-level education have exhibited more hesitancy than uneducated or highly educated respondents [45,47]. Individuals living with their older family members (60+ years of age), displayed more vaccine hesitancy than those who were not living with the elderly [56].

Furthermore, the regression analyses showed that the likelihood of vaccine hesitancy is almost three-fourths less among the respondents, who strongly agreed or agreed with the items of the ’perceived benefits’ of the COVID-19 vaccine. It shows that awareness of the perceived benefits of the vaccination would reduce the level of vaccine hesitancy. These individuals believed that after getting the vaccine, the risk of getting the COVID-19 infection would be low, lesser risk of severity [57,58]. The results supported the previous studies that emphasized awareness campaigns among individuals [59].

Similarly, the 5C model has also supported the existing literature that psychological antecedents like, ‘*confidence*’ and ‘*collective responsibility*’ are crucial factors in reducing vaccine hesitancy. Those respondents were confident in the safety and the effectiveness of the COVID-19 vaccine, and had a sense of social responsibility, and collective work to reduce the infection in their society [30,33,46,47].

On the contrary, the regression analysis of the TPB model confirmed a four times higher likelihood of vaccine hesitancy among the people who had a negative attitude towards the COVID-19 vaccine. The findings are in tune with the previous studies, which showed a negative attitude is the root cause of vaccine hesitancy in India [24,59,60].

In addition to, the five major groups of barriers of vaccination mentioned by the respondents in the study. This exhibited that almost two-fifths of the total barriers were shared only by socio-economic and demographic reasons (38.9%). This finding has complemented the existing literature that highlights the socio-economic and demographic inequities in vaccination coverage [61,62]. On the other hand, there is also a large section of people, who were hesitant about the COVID-19 vaccine because of their lack of confidence due to the misinformation’ (37.5%) [63,64], and ‘individual health reasons’ (11.1%) [65,66].

The study also showed that respondents had trusted the information given or shared by their family members, friends, relatives, and health workers, had lesser vaccine hesitancy, while, mass media and social media, as a source of information, showed higher vaccine hesitancy. In tune with the previous studies, the findings confirmed the menace of fake news and misinformation created by social media and mass media, and have more trust in their surroundings and local level source information [67–69]. Beyond the findings of the source of information, this study has also highlighted that the majority of people who had ‘no opinion’, and ‘strongly disagreed or disagreed’ about the safety of the vaccine among pregnant women and children (below 18), its level and duration of side-effects, had shown higher vaccine hesitancy, than the respondents, who had strongly agreed or agreed with it [70–72].

## Conclusion

The present study aligns with prior research, identifying specific socio-economic and demographic groups at high risk for vaccine hesitancy. In addition, the study utilized three health behavioral models to identify key determinants of vaccine hesitancy. Among these determinants, the most salient factor was individuals’ attitudes toward the COVID-19 vaccine. Regardless of socio-economic or demographic characteristics, some individuals lacked trust in the vaccine’s effectiveness or considered it unnecessary. Others were not sufficiently concerned about the COVID-19 infection or its potentially fatal consequences. Misinformation and beliefs against COVID-19 and its vaccine also contributed to vaccine hesitancy, reducing individuals’ confidence, and creating negative attitudes towards vaccination [44]. This study underscores the importance of addressing concerns about vaccine efficacy and countering misinformation to encourage vaccine uptake.

To evade vaccine hesitancy at the interpersonal level through behavioral changes, the targeted population (individuals, families, relatives, and community health officials) could be included in the process of diffusion of authentic information. Government officials could introduce effective public education and outreach activities by using multiple platforms like community-level representatives and health officials interactions, radio jingles, hoarding, distinguished persons, m-health, and e-health options, to curb the misinformation, at the local as well as at the mass level [].

From a policy perspective, it would be beneficial for the government to consider taking proactive measures to launch mass awareness campaigns at various levels, including individuals, locals, and communities, to address vaccine hesitancy. Suggestively, policymakers could consider distributing accurate information and messages through mass media platforms and community health workers trained by health officials. They might prioritize addressing the reasons behind people’s negative attitudes by instilling confidence and trust in the vaccine and its process, emphasizing the advantages of vaccination, and raising awareness about civic responsibilities as citizens. Looking ahead, taking precautionary steps like mass vaccination is crucial to mitigate the impact of potential disease outbreaks, similar to the experience with COVID-19.

## Study limitations

This study has certain limitations, which need to be acknowledged. This study is based on the self-reported samples (either unvaccinated or had single doses of vaccine only), during the survey period (July 2022, to September 2022), and no other follow-up and cross-verification was conducted of the samples. Therefore, there could be differences in the vaccination coverage among the selected blocks.

## Supporting information

S1 File List of variables. (DOCX)

S2 File Strobe-statement.org. (DOCX)

S3 File Supporting tables. (DOCX)

S1 Data Data COVID 19 vaccine. (XLS)

## Abbreviations

COVID-19: Coronavirus disease
HBM: Health Belief Model
TPB: Theory of Planned Behavior
