# Supplementary material for: COVID-19 vaccine hesitancy among adults in India: A primary study based on health behavior theories and 5C psychological antecedents model
Source: PLoS One. 2024 May 9;19(5):e0294480. doi: 10.1371/journal.pone.0294480 (PMC11081298; doi:10.1371/journal.pone.0294480)
Supplement: S1 File — (DOCX) [file pone.0294480.s001.docx]

| **List of variables** | | |
| --- | --- | --- |
| **S. No.** | **Name of the variable** | **Code used** |
| 1 | Age of the respondent | respc_age |
| 2 | Sex of the respondent | respondent_sex |
| 3 | Marital status | marital |
| 4 | Caste of the respondent | caste_1 |
| 5 | Religion | relig_new |
| 6 | Education level of the respondent | edu_re |
| 7 | Occupation of the respondent | occr |
| 8 | Family members | family_member |
| 9 | Family member (above 60 years of age) | famem_above60 |
| 10 | Household monthly income | economic_status_low |
| 11 | Knowledge about COVID-19 vaccine: Respondent’s opinion: Side effects of COVID-19 vaccine do not last longer than 2 days. | kcv_2seffec2d |
| 12 | Knowledge about COVID-19 vaccine: Respondent’s opinion: COVID-19 vaccine has very mild side effect. | kcv_2mildeffects |
| 13 | Knowledge about COVID-19 vaccine: Respondent’s opinion: COVID-19 vaccine is safe for children under 18 years old. | kcv_2safe18b |
| 14 | Knowledge about COVID-19 vaccine: Respondent’s opinion: COVID-19 vaccine is safe for pregnant women. | kcv_2safepreg |
| 15 | Knowledge about COVID-19 Vaccine | knowcv |
| 16 | Knowledge about the Vaccination Process: Does respondent know the correct doses of Covid-19 vaccine? | kvp_1cvdose |
| 17 | Knowledge about the Vaccination Process: Does respondent know that health care workers are providing the COVID-19 vaccine at their door step? | kvp_1hwdoor |
| 18 | Knowledge about the Vaccination Process: Does respondent know that COVID-19 vaccine cannot be directly purchased from a pharmacy store? | kvp_1nopharma |
| 19 | Knowledge about the Vaccination Process: Does respondent know that he/she would have to register online to receive the COVID-19 vaccination? | kvp_1regonline |
| 20 | Knowledge about the Vaccination Process: Does respondent know that he/she would have to consult with a doctor to receive the COVID-19 vaccination? | kvp_1consultdoc |
| 21 | Knowledge about the Vaccination Process: Does respondent know that he/she can receive the COVID-19 vaccination from selected health facilities? | kvp_1healthcvac |
| 22 | Knowledge about the COVID-19 vaccine and vaccination process | knowcvpro |
| 23 | Behavioral practices: Conscious about using the sanitizer, handwash, or soap | bpp_knowhandwash |
| 24 | Behavioral practices: Always wear mask when outside home or around people | bpp_wmask |
| 25 | Behavioral practices: Avoid crowds to prevent risk of getting COVID-19 | bpp_avcrowd |
| 26 | Behavioral practices: to prevent COVID-19 | behavprac |
| 27 | Does respondent a health worker? | health_worker |
| 28 | COVID-19 vaccine hesitancy | c_cvhs2 |
| 29 | Tobacco consumption | tobacoo_re |
| 30 | Alcohol consumption | alcohol_re |
| 31 | Perceived susceptibility: Respondent worried about the likelihood of getting infected by COVID-19 | psus_2worriedc |
| 32 | Perceived susceptibility: Respondent was high at risk of COVID-19 because of his/her health conditions. | psus_2risk |
| 33 | Health Belief Model: Perceived susceptibility | mpsusceptibility |
| 34 | Perceived severity: Respondent will be very sick if he/she get infected by COVID-19. | psev_2sickinf |
| 35 | Perceived severity: Respondent was very concerned that he/she could die from COVID-19. | psev_2death |
| 36 | Health Belief Model: Perceived severity | mpseverity |
| 37 | Perceived benefits: Respondent think vaccination is good because it will make him/her less worried about COVID-19. | pben_2rworry |
| 38 | Perceived benefits: Respondent believes vaccination will decrease his/her risk of getting infected by COVID-19 | pben_2rrisk |
| 39 | Perceived benefits: Respondent get vaccinated and then get infected with the Coronavirus, then the COVID-19 complications will decrease. | pben_2rsever |
| 40 | Health Belief Model: Perceived benefits | mpbenefits |
| 41 | Perceived barriers: Respondent were worried that the possible side effects of the COVID-19 vaccination would interfere with his/her usual activities | pbar_2sideeffects |
| 42 | Perceived barriers: Respondent were concerned about the efficacy of the COVID-19 vaccine. | pbar_2weffic |
| 43 | Perceived barriers: Respondent was concerned that he/she may receive faulty/fake COVID-19 vaccine. | pbar_2fakevac |
| 44 | Perceived barriers: Respondent concerned that the development of a COVID-19 vaccine is too rushed to test its safety properly. | pbar_2imptrial |
| 45 | Perceived barriers: Respondent concerned about the long-term side effects of the COVID-19 vaccination. | pbar_2seffects |
| 46 | Health Belief Model: Perceived barriers | mpbarriers |
| 47 | Cues to action: Respondents got infected with COVID-19 | c2a_resinfect1 |
| 48 | Cues to action: A family member got infected with COVID-19 | c2a_fmeminfect2 |
| 49 | Cues to action: social media (e.g., Facebook) or online news portals/blogs as the source of knowledge | c2a_smedia3 |
| 50 | Cues to action: Print media as the source of knowledge about COVID-19 vaccine | c2a_prmedia4 |
| 51 | Attitude towards vaccine: Respondent think the COVID-19 vaccine probably will not work. | atv_2ineffctvac |
| 52 | Attitude towards vaccine: Respondent do not trust the COVID-19 vaccine | atv_2notrustvac |
| 53 | Attitude towards vaccine: Respondent think the COVID-19 vaccine is unnecessary. | atv_2unecesvac |
| 54 | Attitude towards vaccine: Respondent think it is not important to get a vaccine to protect people from the COVID-19. | atv_2unimportvac |
| 55 | Attitude towards vaccine: Respondent do not need a COVID-19 vaccine because you are healthy and at low risk for infection. | atv_2noneedvac |
| 56 | Attitude towards vaccine: Respondent do not need a COVID-19 vaccine because even if he/she get infected, he/she will not become seriously ill. | atv_2nosevrill |
| 57 | Attitude towards COVID-19 vaccine | attitude_cv |
| 58 | Subjective norm: believe on family members will support you to get the COVID-19 vaccine | sn_2fsupvac |
| 59 | Perceived behavior control: if he/she can register for the COVID-19 vaccination | pbc_2regstrvac |
| 60 | Anticipated regret: If respondent do not get a COVID-19 vaccine and end up getting Coronavirus, he/she will regret not getting the vaccination | ar_2regret |
| 61 | 5C: Confidence: Respondent is completely confident that COVID-19 vaccines are safe | con5c_2safevac |
| 62 | 5C: Confidence: Respondent is completely confident that COVID-19 vaccines are effective. | con5c_2effctvac |
| 63 | 5C: Constraints: Everyday work stress prevents the respondents from getting vaccinated against COVID-19. | cons5c_2stresuvac |
| 64 | 5C: Constraints: Respondent feels inconvenient to receive COVID-19 vaccine. | cons5c_2inconvac |
| 65 | 5C: Constraints: Visiting the doctor’s place makes the respondent uncomfortable; this keeps hime/her from getting vaccinated against COVID-19. | cons5c_2worryvdoc |
| 66 | 5C: Complacency: Respondent think it is unnecessary to get vaccinated as it cannot prevent COVID-19. | com5c_2unnecessvac |
| 67 | 5C: Complacency: Respondent believe on his/her immune system is powerful; it will protect from COVID-19. | com5c_2strimmun |
| 68 | 5C: Complacency: Respondent believes that COVID-19 is not much a severe disease that he/she should get vaccinated against it. | com5c_2cnserious |
| 69 | 5C: Calculation: Respondent think about getting vaccinated against COVID-19, and weigh the benefits and risks to make the best decision possible. | cal5c_2wdecision |
| 70 | 5C: Calculation: Respondent think about getting vaccinated against COVID-19, he/ she will first consider whether it is effective or not. | cal5c_2coneffectvac |
| 71 | 5C: Calculation: Respondent get COVID-19 vaccine, he/she need to know about this vaccine in detail | cal5c_2vacdetail |
| 72 | 5C: Collective Responsibility: Respondent will take the COVID-19 vaccine because, in that way, he/she can protect people with a weaker immune system | cr5c_2protectp |
| 73 | 5C: Collective Responsibility: Respondent will think vaccination against COVID-19 is a collective action to prevent the spread of diseases. | cr5c_2caction |
| 74 | 5C: Collective Responsibility: If everyone is vaccinated, he/she don't need to get vaccinated | cr5c_1nneedvac |
| 75 | 5C antecedents: Confidence | confidence |
| 76 | 5C antecedents: Constraints | constraints |
| 77 | 5C antecedents: Complacency | complacency |
| 78 | 5C antecedents: Calculation | calculation |
| 79 | 5C antecedents: Collective responsibility | cresponsibility |
| 80 | Respondent heard about the COVID-19 | heard_1cvaccine |
| 81 | Source of information about COVID-19 vaccine- social media | sourcei1_1socialm |
| 82 | Source of information about COVID-19 vaccine- mass media | sourcei2_1massm |
| 83 | Source of information about COVID-19 vaccine- family member | sourcei3_1family |
| 84 | Source of information about COVID-19 vaccine- friends or colleagues | sourcei4_1friendsneigh |
| 85 | Source of information about COVID-19 vaccine- relatives | sourcei5_1relatives |
| 86 | Source of information about COVID-19 vaccine- health workers | sourcei6_1healthworker |
| 87 | Source of information about COVID-19 vaccine- others | sourcei7_1others |
| 88 | Respondent's family or friends think of getting COVID-19 vaccine, what respondent will do? | cvsug_1family |
| 89 | Plan to COVID-19 vaccine | cplan_1vacc |
| 90 | Perceived health status | prcvd_1rhealth |
| 91 | Conspiracy belief: People are misled about effectiveness of vaccines | consb_2vacceff |
| 92 | Conspiracy belief: Vaccination can lead to COVID infection | consb_2vacinfec |
| 93 | Major reasons for not taking the COVID-19 vaccine | reason_1domain |
