# Supplementary material for: COVID-19 vaccine hesitancy among adults in India: A primary study based on health behavior theories and 5C psychological antecedents model
Source: PLoS One. 2024 May 9;19(5):e0294480. doi: 10.1371/journal.pone.0294480 (PMC11081298; doi:10.1371/journal.pone.0294480)
Supplement: S2 File — (DOCX) [file pone.0294480.s002.docx]

STROBE Statement—checklist of items that should be included in reports of observational studies

|  | Item No. | Recommendation | Page  No. | Relevant text from manuscript |
| --- | --- | --- | --- | --- |
| **Title and abstract** | 1 | (*a*) Indicate the study’s design with a commonly used term in the title or the abstract | 2 | COVID-19 vaccine hesitancy among adults in India: a **primary study based** on health behavior theories and 5C psychological antecedents model |
|  |  | (*b*) Provide in the abstract an informative and balanced summary of what was done and what was found | 2 | Despite the significant success of India's COVID-19 vaccination program, a sizeable proportion of the adult population remains unvaccinated or has received a single dose of the vaccine. Despite the recommendations of the Government of India for the two doses of the COVID-19 vaccine and the precautionary booster dose, many people were still hesitant towards the COVID-19 full vaccination. Hence, this study aimed to identify the primary behavioral and psychological factors contributing to vaccine hesitancy. **Cross-sectional data was collected via a multi-stage sampling design** by using a scheduled sample survey in the Gorakhpur district of Uttar Pradesh, India, between 15 July 2022 to 30 September 2022. This study has utilized three health behavior models - the Health Belief Model (HBM), the Theory of Planned Behavior (TPB), and the 5C Psychological Antecedents of vaccination, and employed bivariate and multivariable binary logistic regression model to assess the level of vaccine hesitancy and predictive health behavior of the respondents**. Results indicate that among the constructs of the HBM and 5C Antecedents models, "perceived benefits", "confidence" and "collective responsibility" showed a lesser likelihood of COVID-19 vaccine hesitancy. However, in the TPB model constructs, a ‘negative attitude towards the vaccine’ showed a four times higher likelihood of COVID-19 vaccine hesitancy**. From the future policy perspective, this study suggested that addressing the issue of ‘negative attitudes towards the vaccine’ and increasing the trust or confidence for the vaccine through increasing awareness about the benefits of the vaccination in India may reduce vaccine hesitancy. |
| Introduction | | | |  |
| Background/rationale | 2 | Explain the scientific background and rationale for the investigation being reported | 6 | These models are not much explored in the Indian health behavior context, especially for the COVID-19 vaccine [25,51,52]. Additionally, India is one of the largest populated countries at the global level [57], and among its states, Uttar Pradesh (U.P.) shared the largest contribution to the overall population [52]. Furthermore, the U.P. has achieved remarkable success in the government-driven COVID-19 vaccination awareness and campaigns, among all 75 districts. Gorakhpur district, which was among the top five districts in the initial phases of vaccination, coverage, is emerging as a significant district at the state as well as national levels [51]. To represent the rural as well as urban population with socio-economic, and demographic dynamic characteristics, Gorakhpur district was taken as the study area [25]. |
| Objectives | 3 | State specific objectives, including any prespecified hypotheses | 6-7 | From, a future perspective, this study is crucial to understanding the level of vaccine hesitancy and behavioral determinants by taking it as an example for better preparedness for any short-term variations in the COVID-19 infections, or any other disease outbreak in the near future. Therefore, this study has aimed to analyse the level of COVID-19 vaccine hesitancy by the background characteristics of the population and to understand the behavioral and psychological factors of vaccine hesitancy, by using the HBM, TPB, and 5C antecedents’ models. |
| Methods | | | |  |
| Study design | 4 | Present key elements of study design early in the paper | 7 | The vaccine hesitancy study is a population-based cross-sectional study that collected data from the two blocks (i.e., ‘Charganwa’ and ‘Bhathat’) of the Gorakhpur district of Uttar Pradesh (U.P.), India, between July 2022 and September 2022. According to the Census of India (2011), the total population of Gorakhpur district was 4,440,895, and among them, men and women were 51.29% and 48.71%, respectively. Almost four-fifths of the total population resided in rural households (81.17%), and 18.82% in urban households. Further, at the administrative level, Gorakhpur is subdivided into 7 sub-divisions (tehsils), 19 development blocks, and 84 villages. Among them, the Gorakhpur sub-division has 32 villages (the highest number of villages), and almost two-fifths (44.35%) of the Gorakhpur sub-division population is urban, as compared to other tehsils. Further, among the five blocks of the Gorakhpur sub-division, the ‘Charganwa’ block represented the ‘highest’ coverage area for the COVID-19 vaccine, and the ‘Bhathat’ block represented the ‘lowest’ coverage area for the COVID-19 vaccination, in the Gorakhpur district. This low coverage was more prominent among the rural households, while high coverage was represented by the urban households. respectively (data was taken from the block administrative officer). |
| Setting | 5 | Describe the setting, locations, and relevant dates, including periods of recruitment, exposure, follow-up, and data collection | 8 | In the four stages of the multi-stage cross-sectional sampling method, the 600 adult respondents (≥18 years of age) were interviewed between July 15, 2022, and September 30, 2022. In stage 1, the entire Gorakhpur district was subdivided into seven tehsils, and one tehsil (Gorakhpur Sadar) was selected, which has almost equal representation of the rural and urban population. In Stage 2: Among the five blocks of the Gorakhpur Sadar, two blocks were selected as the lowest and highest COVID-19 vaccine coverage area, at the time of the survey. In stage 3: the villages were stratified by the village population size, and the stratum of large and small villages was done as per the census data. Lastly, in Stage 4, villages were selected by a simple random sampling method from each stratum. In each village, a total of adult men and women who were not vaccinated or had a single dose of vaccination were identified with the help of the local health care worker or ANM. A detailed description of the study was shared in the local (Hindi) language, and consent was obtained before the interview. Those, who agreed and provided their consent were included in this study.  Those who were fully vaccinated (had two or more doses of the COVID-19 vaccine) were not included in this survey. The sample size was calculated, by using the following formula: n=(Z_α^2*p*q*(1+R)*(deff))/d^2  Where n denotes the estimated sample size (600 samples); α = the level of statistical significance that was set at 0.05; Zα = the z value at 95% confidence level, (Here, zα=1.96, with 95% confidence level); d denotes the margin of error [54]. Here, d=.05; p is the prevalence of vaccine hesitancy to be 34.3%; q=1-p (q=65.7%); R response rate (Here, R=.1); deff denotes the design effect (here, deff is 1.5). |
| Participants | 6 | (*a*) *Cohort study*—Give the eligibility criteria, and the sources and methods of selection of participants. Describe methods of follow-up  *Case-control study*—Give the eligibility criteria, and the sources and methods of case ascertainment and control selection. Give the rationale for the choice of cases and controls  ***Cross-sectional study*—Give the eligibility criteria, and the sources and methods of selection of participants** | 8 | Where n denotes the estimated sample size (600 samples); α = the level of statistical significance that was set at 0.05; Zα = the z value at 95% confidence level, (Here, zα=1.96, with 95% confidence level); d denotes the margin of error [54]. Here, d=.05; p is the prevalence of vaccine hesitancy to be 34.3%; q=1-p (q=65.7%); R response rate (Here, R=.1); deff denotes the design effect (here, deff is 1.5).  The data was collected by using ‘The Kobo Toolbox Platform’ which is available in the public domain. The quality of the data, real-time, and location were continuously checked through its online connectivity at the website://www.kobotoolbox.org/. The ethical clearance were given by the authors' institute, in New Delhi, India. Ethical approvals were sought from both authors' institutes. |
|  |  | (*b*) *Cohort study*—For matched studies, give matching criteria and number of exposed and unexposed  *Case-control study*—For matched studies, give matching criteria and the number of controls per case |  |  |
| Variables | 7 | Clearly define all outcomes, exposures, predictors, potential confounders, and effect modifiers. Give diagnostic criteria, if applicable | 9-10 | The structured questionnaire has focused on the major components of the HBM, TPB, and 5C psychological antecedents models, along with the socioeconomic and demographic details of the respondents. The questionnaire was divided into two major parts ‘A’ and ‘B’. Part A collected the information of the respondents, while Part B was further divided into eight sections, which collected the study-related information covered under multiple eight (8) sections. Section 1 dealt with the participant’s socioeconomic and demographic characteristics, Section 2 collected information on the COVID-19-related history (disease/ infections/ deaths); Section 3 related to the ‘Knowledge or perception about the COVID-19 vaccine and vaccine hesitancy’; and Section 4 and 5 asked about the respondent’s ‘health status’. In addition, to understand the respondent’s attitude, subjective norms, perceived belief and behavior, and anticipated regret, this study has included the components of the ‘Health Belief Model’, ‘Theory of Planned Behavior’, and ‘The 5C psychological antecedents of vaccination’, in section 5, 6, and 7, respectively. However, the 8th Section deals with the ‘Knowledge and Belief regarding the COVID-19 vaccination.’  In the HBM section, items were included based on the five components of the model, i.e., ‘perceived susceptibility’ ‘perceived severity (∝ =0.781),’, ‘perceived benefits (∝ =0.788)’, ‘perceived barriers (∝ =0.626)’, and ‘cues to action’. Except for ‘Cues to Action’, the rest of the components were rated on a five-point ‘Likert Scale’ ranging from ‘Strongly Disagree’ (1) ‘Disagree’ (2), ‘Can’t Say Anything’ (3), ‘Agree’ (4), ‘Strongly Agree’ (5). Whereas, ‘Cues to Action’ was dichotomized into ‘Yes’ or ‘No’. Furthermore, in the TPB model, the responses ratings were given on the five-point Likert scale (‘Strongly Disagree’, ‘Disagree’, ‘Can’t say Anything’, ‘Agree’, and ‘Strongly Agree’) for each item of the four components of TPB: ‘negative attitude towards vaccine’ (∝ =0.781), ‘subjective norm’, ‘perceived behavioral control’, and ‘anticipated regret’. Similarly, in the 5C psychological antecedents of the COVID-19 vaccination model, the same ratings were used to measure the 14 items of the five components of 5C: (a) Confidence (∝ =0.844), (b) Constraints, (c) Complacency, (∝ =0.637), (d) calculation, (∝ =0.864, and (5) Collective Responsibility (∝ =0.637). A detailed summary of the items in these three models has been given in the supplementary S1 Table, along with their reliability coefficient for each component.  To measure vaccine hesitancy, ‘Question (3.8): Are you planning to take the COVID-19 vaccine that is currently available?’ was asked to the respondents, with five-point Likert scale response, i.e., ‘Definitely’, ‘Probably’, ‘Not sure’, ‘Probably not’, and ‘Definitely not’. Among the five-point Likert scales, ‘Definitely’ and ‘Probably’ responses were considered non-hesitant responses, on the other hand, ‘Not Sure’, ‘Probably not’, and ‘Definitely not’ responses were considered vaccine-hesitant. |
| Data sources/ measurement | 8* | For each variable of interest, give sources of data and details of methods of assessment (measurement). Describe comparability of assessment methods if there is more than one group |  |  |
| Bias | 9 | Describe any efforts to address potential sources of bias | 10-11 |  |
| Study size | 10 | Explain how the study size was arrived at | 8 | Those who were fully vaccinated (had two or more doses of the COVID-19 vaccine) were not included in this survey. The sample size was calculated, by using the following formula: n=(Z_α^2*p*q*(1+R)*(deff))/d^2  Where n denotes the estimated sample size (600 samples); α = the level of statistical significance that was set at 0.05; Zα = the z value at 95% confidence level, (Here, zα=1.96, with 95% confidence level); d denotes the margin of error [54]. Here, d=.05; p is the prevalence of vaccine hesitancy to be 34.3%; q=1-p (q=65.7%); R response rate (Here, R=.1); deff denotes the design effect (here, deff is 1.5). |

Continued on next page

| Quantitative variables | 11 | Explain how quantitative variables were handled in the analyses. If applicable, describe which groupings were chosen and why | 11 | Descriptive statistics were used to analyze the socioeconomic, and demographic characteristics and knowledge of COVID-19 and its vaccine. The sample distribution and percentage for the background characteristics were measured by the mean with standard deviation (SD). However, due to the skewed nature of the samples among the ‘age’ and ‘households’ members’, the median with SD was calculated to know the distribution. However, bivariate analyses were used to estimate the level of vaccine hesitancy based on background characteristics, knowledge level about COVID-19 vaccination, and intention of getting COVID-19 vaccination. Moreover, the Chi-square test was used to compare the observed results with the expected results, with p-values (two-tailed with a significance level of 5%). Similarly, the bivariate analyses were performed for the three models (HBM, TPB, and 5C), separately, to assess the level of COVID-19 vaccine hesitancy by the items of the models. The details of the outcome variable, i.e., vaccine hesitancy are mentioned above. The multivariable binary logistic regression model was employed to predict the association between the level of COVID-19 vaccine hesitancy and the major components of the HBM, TPB, and 5C psychological antecedent models. All three health behavior models were analyzed separately, by using the STATA-15 software [55]. |
| --- | --- | --- | --- | --- |
| Statistical methods | 12 | (*a*) Describe all statistical methods, including those used to control for confounding | 11 | The sample distribution and percentage for the background characteristics were measured by the mean with standard deviation (SD). However, due to the skewed nature of the samples among the ‘age’ and ‘households’ members’, the median with SD was calculated to know the distribution. However, bivariate analyses were used to estimate the level of vaccine hesitancy based on background characteristics, knowledge level about COVID-19 vaccination, and intention of getting COVID-19 vaccination. Moreover, the Chi-square test was used to compare the observed results with the expected results, with p-values (two-tailed with a significance level of 5%). Similarly, the bivariate analyses were performed for the three models (HBM, TPB, and 5C), separately, to assess the level of COVID-19 vaccine hesitancy by the items of the models. The details of the outcome variable, i.e., vaccine hesitancy are mentioned above. The multivariable binary logistic regression model was employed to predict the association between the level of COVID-19 vaccine hesitancy and the major components of the HBM, TPB, and 5C psychological antecedent models. |
|  |  | (*b*) Describe any methods used to examine subgroups and interactions | 11 |  |
|  |  | (*c*) Explain how missing data were addressed | 11 |  |
|  |  | (*d*) *Cohort study*—If applicable, explain how loss to follow-up was addressed  *Case-control study*—If applicable, explain how matching of cases and controls was addressed  *Cross-sectional study*—If applicable, describe analytical methods taking account of sampling strategy | 11 |  |
|  |  | (*e*) Describe any sensitivity analyses | 11 |  |
| Results | | | | |
| Participants | 13* | (a) Report numbers of individuals at each stage of study—eg numbers potentially eligible, examined for eligibility, confirmed eligible, included in the study, completing follow-up, and analysed | 13 | Table 1 indicates the sample distribution and the percentage of COVID-19 vaccine hesitancy based on the socio-economic characteristics of the selected respondents (N=600) in this study. In this study, 524 respondents (87.3%) had one dose of COVID-19, while, 76 respondents (12.7%) were unvaccinated at the time of this survey (July 2022 to September 2022). Almost half (n = 304) of the respondents were female (50.7%). Most of the respondents (185) belonged to the 18-24 years age group (30.8%), followed by the 25-34 years age group (n = 139, 23.2%), and the 35-44 years age group (n =107, 17.8%), while, 55 and above years age group had the least representation in this survey (n=78, 13%). The median age of the samples was 32 years ± 14.73. Almost, 431 (71.8%) respondents were married, and 169 respondents (28.2%) were unmarried during the survey. |
|  |  | (b) Give reasons for non-participation at each stage |  |  |
|  |  | (c) Consider use of a flow diagram |  |  |
| Descriptive data | 14* | (a) Give characteristics of study participants (eg demographic, clinical, social) and information on exposures and potential confounders | 13 | The study showed that 32.9% of unvaccinated people were hesitant about the COVID-19 vaccine compared to those who had a single dose of the COVID-19 vaccine (9%) (Table 1). Moreover, by socio-economic and demographic characteristics, older (65+) people (27.3%), men (13.2%), married persons (12.5%), below primary level educated (21.2%), and those with no education (18%), employed in the formal sector (27.2%) had a comparatively higher hesitancy for the COVID-19 vaccine than their counterparts. Similarly, amongst the social groups, respondents belonging to the general caste (21.2%), and having older family members (16.1%,), had higher hesitancy than the other caste groups. Similarly, respondents who were regularly consuming alcohol (15.9%) and tobacco (14.2%) (in any form) showed higher vaccine hesitancy than their counterparts. |
|  |  | (b) Indicate number of participants with missing data for each variable of interest | 13 |  |
|  |  | (c) *Cohort study*—Summarise follow-up time (eg, average and total amount) |  |  |
| Outcome data | 15* | *Cohort study*—Report numbers of outcome events or summary measures over time |  |  |
|  |  | *Case-control study—*Report numbers in each exposure category, or summary measures of exposure | *15-16* |  |
|  |  | *Cross-sectional study—*Report numbers of outcome events or summary measures | 13 | Almost 92% of respondents mentioned that they had heard about the COVID-19 vaccine through multiple sources of information. Among them, the major sources of information were family members (81.90%), mass media (56.60%), friends and neighbours (55%), nearest relatives (46.10%), social media (41.20%), and health workers (37.60%), consecutively. In Fig 1B, by the source of information on the COVID-19 vaccine, those who had information from, a friend or neighbour (7.2%), or relatives (5.1%), showed significantly less vaccine hesitancy than those who did not have it (S3 Table). |
| Main results | 16 | (*a*) Give unadjusted estimates and, if applicable, confounder-adjusted estimates and their precision (eg, 95% confidence interval). Make clear which confounders were adjusted for and why they were included | 15-20 | Vaccine hesitancy was reported among 12% of the total respondents (N=600). In addition, Table 5 indicates the results for the level of predictability of the association between COVID-19 vaccine hesitancy through the health behavior models (HBM, TPB, and 5C Psychological Antecedents, separately). It revealed that among all the five constructs of the HBM (perceived susceptibility, perceived severity, perceived benefits, perceived barriers, and cues to action), only the ‘perceived benefits’ showed a significant negative association with COVID-19 vaccine hesitancy [Adjusted odds ratio: 0.26, with 95% C.I.: 0.15-0.47].  Similarly, in the TPB model, those who had a higher negative attitude towards the COVID-19 vaccine were four times more likely to have hesitancy than those who didn’t have a less negative attitude towards the vaccine [Adjusted odds ratio: 4.17, with 95% C.I.: 2.06-8.45].  Furthermore, in the 5C model, ‘confidence’ [Adjusted odds ratio: 0.42, with 95% C.I.: 0.24-0.72], and ‘collective responsibility’ [Adjusted odds ratio: 0.48, with 95% C.I.: 0.27-0.89] components showed a significant negative association with COVID-19 vaccine hesitancy. In other words, it could suggest that a significant increase in the confidence of people in the COVID-19 vaccination, and their feeling of responsibility for society to prevent the spread of disease reduced COVID-19 vaccine hesitancy. |
|  |  | (*b*) Report category boundaries when continuous variables were categorized |  |  |
|  |  | (*c*) If relevant, consider translating estimates of relative risk into absolute risk for a meaningful time period |  |  |

Continued on next page

| Other analyses | 17 | Report other analyses done—eg analyses of subgroups and interactions, and sensitivity analyses | 31 | Supporting information file |
| --- | --- | --- | --- | --- |
| Discussion | | | | |
| Key results | 18 | Summarise key results with reference to study objectives | 22 | The objective of this study was to assess the level of COVID-19 vaccine hesitancy and identify the major determinants of health behaviors among the adult population of Gorakhpur district, Uttar Pradesh, India [54]. The results showed approximately 12% vaccine hesitancy among the selected participants, who were unvaccinated or had only received a single dose of the COVID-19 vaccine, at the time of this survey. This study identified the major predictive health behavior for COVID-19 vaccine hesitancy by using the three health behavior models. This study has also identified the major barriers mentioned by the respondents to getting the COVID-19 vaccines.  The findings of the study exhibited that vaccine hesitancy was higher among older individuals (> 40 years of age), mostly among married individuals, than their respective counterparts. Similarly, individuals working in the formal sector showed more vaccine hesitancy than students and employees in the informal sector [64,65]. On the contrary, respondents with below primary-level education have exhibited more hesitancy than uneducated or highly educated respondents [61]. Individuals living with their older family members (60+ years of age), displayed more vaccine hesitancy than those who were not living with the elderly [65,68]. |
| Limitations | 19 | Discuss limitations of the study, taking into account sources of potential bias or imprecision. Discuss both direction and magnitude of any potential bias | 25 | This study has certain limitations, which need to be acknowledged. This study is based on the self-reported samples (either unvaccinated or had single doses of vaccine only), during the survey period (July 2022, to September 2022), and no other follow-up and cross-verification was conducted of the samples. Therefore, there could be differences in the vaccination coverage among the selected blocks. |
| Interpretation | 20 | Give a cautious overall interpretation of results considering objectives, limitations, multiplicity of analyses, results from similar studies, and other relevant evidence | 22-23 | The study also showed that respondents had trusted the information given or shared by their family members, friends, relatives, and health workers, had lesser vaccine hesitancy, while, mass media and social media, as a source of information, showed higher vaccine hesitancy. In tune with the previous studies, the findings confirmed the menace of fake news and misinformation created by social media and mass media, and have more trust in their surroundings and local level source information [67-69]. Beyond the findings of the source of information, this study has also highlighted that the majority of people who had ‘no opinion’, and ‘strongly disagreed or disagreed’ about the safety of the vaccine among pregnant women and children (below 18), its level and duration of side-effects, had shown higher vaccine hesitancy, than the respondents, who had strongly agreed or agreed with it [70-72]. |
| Generalisability | 21 | Discuss the generalisability (external validity) of the study results | 24 |  |
| Other information | |  | | |
| Funding | 22 | Give the source of funding and the role of the funders for the present study and, if applicable, for the original study on which the present article is based |  | This study is funded by the Indian Council of Medical Research (ICMR), New Delhi. |

*Give information separately for cases and controls in case-control studies and, if applicable, for exposed and unexposed groups in cohort and cross-sectional studies.

**Note:** An Explanation and Elaboration article discusses each checklist item and gives methodological background and published examples of transparent reporting. The STROBE checklist is best used in conjunction with this article (freely available on the Web sites of PLoS Medicine at http://www.plosmedicine.org/, Annals of Internal Medicine at http://www.annals.org/, and Epidemiology at http://www.epidem.com/). Information on the STROBE Initiative is available at www.strobe-statement.org.
