# Supplementary material for: COVID-19 vaccine hesitancy among adults in India: A primary study based on health behavior theories and 5C psychological antecedents model
Source: PLoS One. 2024 May 9;19(5):e0294480. doi: 10.1371/journal.pone.0294480 (PMC11081298; doi:10.1371/journal.pone.0294480)
Supplement: S3 File — (DOCX) [file pone.0294480.s003.docx]

| S1 Table. Items used to measure HBM, TPB, and 5C Psychological antecedents of vaccination | |  |
| --- | --- | --- |
| **Statements** | **Cronbach α** | |
| **The Health Belief Model** |  | |
| **Perceived susceptibility** | NA | |
| Respondent is at higher risk of COVID-19 because of his/her health conditions. |  | |
| **Perceived severity** | 0.781 | |
| Respondent think that he/she will be very sick if get infected by COVID-19. |  | |
| Respondent is very concerned that he/she could die from COVID-19. |  | |
| **Perceived benefits** | 0.778 | |
| Respondent thinks that vaccination is good because it will make less worried about COVID-19. |  | |
| Respondent believes that vaccination will decrease the risk of getting infected by COVID-19. |  | |
| Respondent thinks the complications of COVID-19 will decrease if he/she get vaccinated and then get infected with the Coronavirus. |  | |
| **Perceived barriers** | 0.626 | |
| Respondent is worried that the possible side-effects of the COVID-19 vaccination would interfere with his/her usual activities. |  | |
| Respondent is concerned about the efficacy of the COVID-19 vaccine. |  | |
| Respondent has a concern that they may receive faulty/ fake COVID-19 vaccine. |  | |
| It concerns the Respondent that the development of a COVID-19 vaccine is too rushed to test its safety properly. |  | |
| Respondent has concerned about the long-term side-effects of the COVID-19 vaccination. |  | |
| **Cues to Action** | NA | |
| Social media (e.g., Facebook) or online news portals/blogs as a source of knowledge about the COVID-19 vaccine. |  | |
| Printed newspaper as a source of knowledge about the COVID-19 vaccine. |  | |
|  |  | |
| **The Theory of Planned Behaviour (TPB)** |  | |
| **Negative attitude towards vaccine** | 0.781 | |
| Respondent thinks the COVID-19 vaccine probably will not work. |  | |
| Respondent doesn't trust the COVID-19 vaccine. |  | |
| Respondent thinks the COVID-19 vaccine is unnecessary. |  | |
| Respondent thinks that it is not important to get a vaccine to protect people from the COVID-19. |  | |
| Respondent does not need a COVID-19 vaccine because he/she is healthy and at low risk for infection. |  | |
| Respondent does not need a COVID-19 vaccine because even if he/she get infected, they will not become seriously ill. |  | |
| **Subjective norm** | NA | |
| Respondent believes that his/her family members will support him/her to get vaccinated against COVID-19. |  | |
| **Perceived behavioral control** | NA | |
| If respondent want, he/she can register for COVID 19 vaccination. |  | |
| **Anticipated regret** | NA | |
| If respondent does not get a COVID-19 vaccine and end up getting Coronavirus, he/she will regret not getting the vaccination. |  | |
|  |  | |
| **The 5C Psychological Antecedents of Vaccination** |  | |
| **Confidence** | 0.8441 | |
| Respondent is completely confident that COVID-19 vaccines are safe. |  | |
| Respondent is completely confident that COVID-19 vaccines are effective. |  | |
| **Constraints** | NA | |
| Everyday work stress may prevent the respondent from getting vaccinated. |  | |
| **Complacency** | 0.637 | |
| Respondent thinks that it is unnecessary to receive vaccinations as it cannot prevent COVID-19. |  | |
| Respondent believe that his/her immune system is powerful; it will protect him/her from COVID-19. |  | |
| Respondent believe COVID-19 is not much a severe disease that he/she should get vaccinated against it. |  | |
| **Calculation** | 0.864 | |
| When respondent thinks about getting vaccinated against COVID 19, he/she weigh the benefits and risks to make the best decision possible. |  | |
| When respondent thinks about getting vaccinated against COVID 19, he/she will first consider whether it is effective or not. |  | |
| Before get COVID-19 vaccinated, respondent need to know about this vaccine in details. |  | |
| **Collective responsibility** | 0.720 | |
| Respondent will take COVID 19 vaccine because, in that way, he/she can protect people with a weaker immune system. |  | |
| Respondent think vaccination against COVID 19 is a collective action to prevent the spread of diseases. |  | |

| S2 Table. Prevalence of COVID-19 by the respondent’s socio-economic characteristics | | |
| --- | --- | --- |
| Variables | Study sample (n) | Prevalence of COVID-19 (%) |
| Respondent ever been sick with COVID-19 |  |  |
| No | 592 | 98.7 |
| Yes | 8 | 1.3 |
| Any family member ever been sick with COVID-19 |  |  |
| No | 592 | 98.7 |
| Yes | 8 | 1.3 |
| Any relative ever been sick with COVID-19 |  |  |
| No | 580 | 96.7 |
| Yes | 20 | 3.3 |
| Any friend/ neighbour/ colleague ever been sick with COVID-19 |  |  |
| No | 564 | 94 |
| Yes | 36 | 6 |
| Death of any family member due to COVID-19, in last 2 years |  |  |
| No | 597 | 99.5 |
| Yes | 3 | 0.5 |
| Death of any relative due to COVID-19, in last 2 years |  |  |
| No | 599 | 99.8 |
| Yes | 1 | 0.2 |
| Death of any friend/colleague/neighbour/others in social network, due to COVID-19 in last 2years |  |  |
| No | 586 | 97.7 |
| Yes | 14 | 2.3 |
| Total | 600 | 100 |

| S3 Table. Descriptive statistics of the respondent's knowledge about COVID-19 vaccine | | |
| --- | --- | --- |
| Variables | Sample (n) | Knowledge (%) |
| **Have you heard about the COVID-19 vaccine (Yes)** | **553** | **92.20** |
| Source of information from social media (Yes) | 228 | 41.20 |
| Source of information from mass media | 313 | 56.60 |
| Source of information from family members (Yes) | 453 | 81.90 |
| Source of information from friends/neighbour (Yes) | 304 | 55.00 |
| Source of information from relatives (Yes) | 255 | 46.10 |
| Source of information from health care worker (Yes) | 208 | 37.60 |
| **Knowledge of doses of the COVID-19 vaccine** |  |  |
| 1 or 2 | 261 | 47.2 |
| 3 or more than 3 | 255 | 46.1 |
| Don't know | 37 | 6.7 |
| **Knowledge of COVID -19 vaccine effective** |  |  |
| 3-months | 88 | 15.91 |
| more than 3 months | 84 | 15.19 |
| Don't know | 381 | 68.9 |
| **Believe of vaccine can control COVID-19** |  |  |
| No | 28 | 5.10 |
| Yes | 404 | 73.10 |
| Don't know/not sure | 121 | 21.90 |
| **Think side effect may arise after vaccination** |  |  |
| No | 94 | 17.00 |
| Yes | 351 | 63.50 |
| Don't know/not sure | 108 | 19.50 |
| **Type of side effect may arise after vaccination** |  |  |
| Primary side effects | 300 | 85.50 |
| Serious side effects | 27 | 7.70 |
| Don't know/not sure | 24 | 6.80 |
| **Perceived health status** |  |  |
| Very Poor/Poor | 48 | 8 |
| Normal | 200 | 33.3 |
| Good/Very good | 352 | 58.7 |
| **Number of Chronic diseases** |  |  |
| None | 489 | 81.5 |
| 1 | 82 | 13.7 |
| 2+ | 29 | 4.8 |
| **Total** | **600** | **100** |

| S4 Table. Descriptive statistics of the knowledge about the COVID-19 vaccine (n=600) | | | | | |
| --- | --- | --- | --- | --- | --- |
| Knowledge about the COVID-19 vaccine (alpha= 0.6862) | Strongly Disagree | Disagree | No opinion | Agree | Strongly Agree |
|  | | | | | |
| Side-effects of COVID-19 vaccine do not last longer than 2 days | 4.50 | 12.30 | 23.70 | 41.20 | 18.30 |
| COVID-19 vaccine has very mild side-effect | 4.50 | 10.00 | 23.80 | 43.70 | 18.00 |
| COVID-19 vaccine is safe for children under 18 years old | 4.70 | 10.80 | 22.50 | 51.00 | 11.00 |
| COVID-19 vaccine is safe for pregnant women | 28.00 | 21.30 | 35.00 | 12.20 | 3.50 |

| S5 Table. Descriptive statistics of the knowledge about the Covid-19 vaccination process (n=600) | | |
| --- | --- | --- |
| **Knowledge about the Vaccination Process 9 (alpha= 0.6983)** | **No** | **Yes** |
| Do you know the correct doses of COVID-19 vaccine? | 14.70 | 85.30 |
| Do you know that healthcare workers are providing the COVID-19 vaccine at your door step? | 30.50 | 69.50 |
| Do you know that COVID-19 vaccine cannot be directly purchased from a pharmacy store? | 32.00 | 68.00 |
| Do you know that you can receive the COVID-19 vaccination from a selected health facility? | 24.50 | 75.50 |
| Do you know that you will have to register online to receive the COVID-19 vaccination? | 42.80 | 57.20 |
| Do you know that you will have to consult with a doctor to receive the COVID-19 vaccination? | 64.30 | 35.70 |

| S6 Table. Descriptive statistics of the behavioral practices to prevent the COVID-19 (n=600) | | | | |
| --- | --- | --- | --- | --- |
| **Behavioral Practices to Prevent COVID-19 (alpha= 0.8866)** | **Never** | **Sometimes** | **Often** | **Regularly** |
| You are conscious about using sanitizer, hand wash, or soap. | 12.70 | 32.80 | 32.80 | 21.70 |
| You always wear a mask when outside of your home or around other people. | 13.80 | 35.30 | 25.00 | 25.90 |
| You avoid crowds as much as possible to prevent your risk of getting COVID-19. | 14.00 | 34.90 | 28.80 | 22.20 |

| S7 Table. Descriptive statistics of the conspiracy belief regarding the COVID-19 vaccine (n=600) | | | | | |
| --- | --- | --- | --- | --- | --- |
| **Conspiracy Belief regarding COVID-19 vaccine (alpha= 0.7301)** | Strongly Disagree | Disagree | No opinion | Agree | Strongly Agree |
| People are misled about the effectiveness of vaccines. | 13.30 | 47.00 | 31.20 | 5.50 | 3.00 |
| Vaccination can lead to COVID infection. | 23.00 | 38.00 | 33.50 | 4.00 | 1.50 |

| S8 Table. Descriptive statistics of the items used to measure the Health Belief Model among 600 samples | | | |
| --- | --- | --- | --- |
| **Variables** | Strongly Disagree/Disagree | No opinion | Agree/Strongly Agree |
| **Perceived Susceptibility** |  |  |  |
| Respondent at high risk of COVID-19 because of his/ her health conditions | 42.7 | 21 | 36.3 |
| **Perceived Severity** |  |  |  |
| Respondent will be very sick if he/she get infected by COVID-19 | 38.7 | 29.8 | 31.5 |
| Respondent was very concerned that he/she could die from COVID-19 | 53.7 | 30.3 | 16 |
| **Perceived Benefits** |  |  |  |
| Respondent think vaccination is good because it will make him/her less worried about COVID-19 | 11.8 | 20.3 | 67.8 |
| Respondent believe vaccination will decrease his/her risk of getting infected by COVID-19 | 7.7 | 21.5 | 70.8 |
| Respondent think the complications of COVID-19 will decrease if he/she get vaccinated and then get infected with the Coronavirus. | 9.8 | 24.3 | 65.8 |
| **Perceived Barriers** |  |  |  |
| Respondent worried that the possible side effects of the COVID-19 vaccination would interfere with his/her usual activities | 26.3 | 35.5 | 38.2 |
| Respondent concerned about the efficacy of the COVID-19 vaccine | 36.7 | 22.5 | 40.8 |
| Respondent have a concern that he/she may receive faulty/fake COVID-19 vaccine | 48.5 | 35.2 | 16.3 |
| It concerns respondent that the development of a COVID-19 vaccine is too rushed to test its safety properly | 49.7 | 30.8 | 19.5 |
| Respondent concerned about the long-term side effects of the COVID-19 vaccination | 44.7 | 24.2 | 31.2 |
|  |  |  |  |
|  | Percentage |  |  |
| **Cues to Action** |  |  |  |
| Social media (e.g., Facebook) or online news portals/blogs as a source of knowledge about the COVID-19 vaccine |  |  |  |
| Yes | 41.8 |  |  |
| No | 58.2 |  |  |
| Printed newspaper as a source of knowledge about the COVID-19 vaccine |  |  |  |
| Yes | 59 |  |  |
| No | 41 |  |  |

| S9 Table. Descriptive statistics of the items used in Theory of Planned Behaviour Model among 600 samples | | | |
| --- | --- | --- | --- |
| **Variables** | Strongly Disagree/Disagree | No opinion | Agree/Strongly Agree |
| **Negative Attitude towards vaccine** | | | |
| Respondent thinks the COVID-19 vaccine probably will not work. | 59.8 | 28.8 | 11.3 |
| Respondent doesn't trust the COVID-19 vaccine | 74.3 | 17.2 | 8.5 |
| Respondent thinks the COVID-19 vaccine is unnecessary | 79.2 | 14.2 | 6.7 |
| Respondent thinks that it is not important to get a vaccine to protect people from the COVID-19 | 74.3 | 17.3 | 8.3 |
| Respondent does not need a COVID-19 vaccine because he/she is healthy and at low risk for infection | 65.2 | 22 | 12.8 |
| Respondent does not need a COVID-19 vaccine because even if he/she get infected, they will not become seriously ill | 62.2 | 25.8 | 12 |
| **Subjective norm** | | | |
| Respondent believes that his/her family members will support him/her to get vaccinated against COVID-19 | 9.2 | 19.2 | 71.7 |
| **Perceived behavioral control** | | | |
| If respondent want, he/she can register for COVID 19 vaccination | 14.5 | 21.3 | 64.2 |
| **Anticipated regret** | | | |
| If respondent does not get a COVID-19 vaccine and end up getting Coronavirus, he/she will regret not getting the vaccination | 8.7 | 14.2 | 77.2 |

| S10 Table. Descriptive statistics for the items used to measure the 5C Psychological antecedents of the vaccination model among the 600 samples | | | |
| --- | --- | --- | --- |
| **Variables** | Strongly Disagree/Disagree | No opinion | Agree/Strongly Agree |
| **The 5C Psychological Antecedents of Vaccination** |  |  |  |
| **Confidence** |  |  |  |
| Respondent is completely confident that COVID 19 vaccines are safe | 8.20 | 26.50 | 65.30 |
| Respondent is completely confident that COVID 19 vaccines are effective | 6.20 | 22.70 | 71.20 |
| **Constraints** |  |  |  |
| Everyday work stress may prevent the respondent from getting vaccinated | 40.00 | 23.30 | 36.70 |
| **Complacency** |  |  |  |
| Respondent thinks that it is unnecessary to receive vaccinations as it cannot prevent COVID-19 | 69.50 | 18.70 | 11.80 |
| Respondent believe that his/her immune system is powerful; it will protect him/her from COVID-19 | 38.20 | 34.00 | 27.80 |
| Respondent believe COVID-19 is not much a severe disease that he/she should get vaccinated against it | 58.80 | 20.20 | 21.00 |
| **Calculation** |  |  |  |
| When respondent thinks about getting vaccinated against COVID 19, he/she weigh the benefits and risks to make the best decision possible | 18.20 | 18.50 | 63.30 |
| When respondent thinks about getting vaccinated against COVID 19, he/she will first consider whether it is effective or not | 16.70 | 18.80 | 64.50 |
| Before get COVID-19 vaccinated, respondent need to know about this vaccine in details | 11.50 | 15.80 | 72.70 |
| **Collective responsibility** |  |  |  |
| Respondent will take COVID 19 vaccine because, in that way, he/she can protect people with a weaker immune system | 9.00 | 28.80 | 62.20 |
| Respondent think vaccination against COVID 19 is a collective action to prevent the spread of diseases | 7.70 | 20.20 | 72.20 |
